# A Two-Stage GMFAMM Approximation for Joint Bias Correction of NASA POWER Hydroclimatic Data: The *ColClim* Web Application

**DOI:** 10.3390/s26134301

**Published:** 2026-07-07

**Authors:** David Arango-Londoño, Delia Ortega-Lenis, Mauricio A. Mazo-Lopera, Paula Moraga

**Affiliations:** 1 Departamento de Estadística, Facultad de Ciencias, Universidad Nacional de Colombia, Sede Medellín, Medellín 050034, Colombia; delia.ortega@javerianacali.edu.co (D.O.-L.); mamazol@unal.edu.co (M.A.M.-L.); 2 Faculty of Engineering and Sciences, Pontificia Universidad Javeriana, Cali 760031, Colombia; 3 Computer, Electrical and Mathematical Sciences and Engineering Division, King Abdullah University of Science and Technology (KAUST), Thuwal 23955-6900, Saudi Arabia; paula.moraga@kaust.edu.sa

**Keywords:** bias correction, satellite data, hydroclimatic modelling, functional additive mixed model, multivariate functional data analysis, NASA POWER, Valle del Cauca, Clausius–Clapeyron, R Shiny, spatial statistics

## Abstract

We propose and empirically evaluate a two-stage approximation to a Generalized Multivariate Functional Additive Mixed Model (GMFAMM) for the joint bias correction of five NASA POWER reanalysis variables: minimum and maximum temperature ($T_{\min}$, $T_{\max}$), relative humidity (RH), solar radiation (Rad), and precipitation occurrence ($P_{\mathrm{bin}}$). Our primary contribution is the first operational-scale evaluation of such a framework (≈200,000 station–day observations, two orders of magnitude beyond previous studies) together with its deployment in an open-access web application. A systematic grid of more than 200 marginal configurations is evaluated on a strict chronological 70/30 hold-out (training 2016–2022; testing 2023–2025) to identify the optimal marginal specification per variable. Against a correctly specified marginal baseline, station-level linear calibration combined with the marginal GAMM removes the bulk of the systematic bias (RMSE reductions of ≈80%, 82% and 30% for $T_{\min}$, $T_{\max}$ and RH). A shared latent step, using the first principal component of the marginal residual matrix as a scalar proxy for $\Lambda_{0}\left(t\right)$, yields additional but *conditional* out-of-sample reductions (≈17% $T_{\max}$, 10% RH, 9% Rad; negligible for $T_{\min}$, with precipitation occurrence retained in the shared representation but its joint gain treated as exploratory); because it requires co-located donor observations, at ungauged locations the deployed pipeline applies the marginal correction only, whose spatial transfer is confirmed by leave-one-station-out cross-validation. The residual cross-correlation structure is consistent with, though not in itself proof of, Clausius–Clapeyron coupling. The trained artefacts are deployed in *ColClim*, an open-access R Shiny application that queries the NASA POWER API and the Open-Meteo forecast service for any location in Colombia and delivers historical bias-corrected series and short-range (1–16 day) forecasts.

## 1. Introduction

Hydroclimatic variables such as temperature, humidity, solar radiation, and precipitation are fundamental drivers of agricultural planning, hydrological risk assessment, and infectious disease surveillance [1]. In tropical regions of South America, the quality and spatial density of ground-based observations are often insufficient for high-resolution operational use [2]. Remote sensing products such as the NASA POWER reanalysis provide spatially continuous estimates at daily temporal resolution, but systematic biases relative to surface observations arising from coarse spatial resolution (≈55 km), cloud–topography interactions, and the Clausius–Clapeyron-driven coupling between temperature and humidity limit their direct applicability [3].

Statistical bias correction of satellite data has been widely studied for individual variables, most often precipitation [4] or temperature [5]. However, the joint nature of hydroclimatic variability means that correcting each variable independently risks destroying the physical correlations among them, which are operationally relevant: an irrigation decision depends jointly on temperature, humidity, and radiation, not on any single variable in isolation. A bias correction framework that preserves and exploits the cross-variable dependence structure is therefore both methodologically and practically desirable.

Multivariate bias correction has been developed extensively in the climate projection literature, where methods such as the *N*-dimensional probability density function transform (MBCn) of Cannon [6] and the Rank Resampling for Distributions and Dependences (R2D2) scheme of Vrac [7] adjust the full joint distribution of several variables, with comparative studies characterising the trade-offs among them [8]. These approaches reorder or resample quantiles so that the corrected ensemble reproduces an observed empirical dependence structure. Our setting differs in two respects. First, the target is a daily reanalysis product evaluated against a sparse ground station network rather than a climate model ensemble, so the operational requirement is correction at arbitrary, possibly ungauged user coordinates rather than over a fixed model grid. Second, we model the cross-variable dependence *generatively*, through a shared latent process embedded in a functional mixed model that also accommodates a binary outcome (precipitation occurrence) alongside continuous ones an outcome-type heterogeneity that quantile-transform methods do not naturally handle.

Functional data analysis provides a natural language for climate time series [9]. Functional Generalized Additive Mixed Models (FGAMMs) have been used for univariate precipitation prediction using ground-based and satellite data [10]. Extending this framework to the multivariate setting requires a latent process architecture that can represent the dominant modes of covariation across heterogeneous outcome types: continuous temperatures and humidity alongside binary precipitation occurrence. The GMFAMM framework of Volkmann et al. [11] provides this architecture, but its validation has been limited to controlled simulation studies and small clinical datasets. This paper provides the first empirical evaluation of a two-stage approximation to the GMFAMM on real-world hydroclimatic data at a dataset scale approximately two orders of magnitude larger than the original framework, together with four methodological adaptations required by this application context.

We propose a two-stage approximation to the GMFAMM that jointly corrects the five NASA POWER reanalysis outputs for the Valle del Cauca region of Colombia. We conduct a systematic evaluation of more than 200 model configurations across distributional families and effect structures, establish the optimal marginal specification for each variable, and quantify against this correctly specified marginal baseline the additional value of the shared latent process. We find that station-level calibration and the marginal GAMM remove most of the systematic bias, while the two-stage shared latent step adds modest, conditional out-of-sample gains (≈17% for $T_{\max}$, ≈10% for RH and ≈9% for solar radiation, with negligible benefit for $T_{\min}$). We further establish spatial generalisation to ungauged locations through leave-one-station-out cross-validation, and describe the deployment of the correction pipeline in *ColClim*, a web-based application that makes bias-corrected hydroclimatic estimates accessible to practitioners without programming skills.

The remainder of this paper is organized as follows. Section 2 describes the study area and data sources, including a summary of the satellite biases documented in the exploratory analysis. Section 3 presents the GMFAMM specification and the methodological adaptations. Section 4 describes the experimental grid and evaluation protocol. Section 5 reports the systematic comparison of marginal models. Section 6 quantifies the added value of joint modelling. Section 7 documents the *ColClim* application. Section 8 discusses limitations and future work.

## 2. Study Area and Data

### 2.1. Study Area

The Valle del Cauca department is located in southwestern Colombia, between $3^{\circ }5^{\prime }$ and $5^{\circ }0^{\prime }$ north latitude and $75^{\circ }41^{\prime }$ and $77^{\circ }33^{\prime }$ west longitude [12]. The inter-Andean valley, at elevations ranging from sea level to 4080 m above sea level, supports an agricultural economy dominated by sugarcane, fruit, and livestock production. The climate follows a bimodal rainfall regime, with peak precipitation in April–May and October–November, and is significantly affected by ENSO teleconnections [13].

### 2.2. Surface Observations

**Figure 1:**
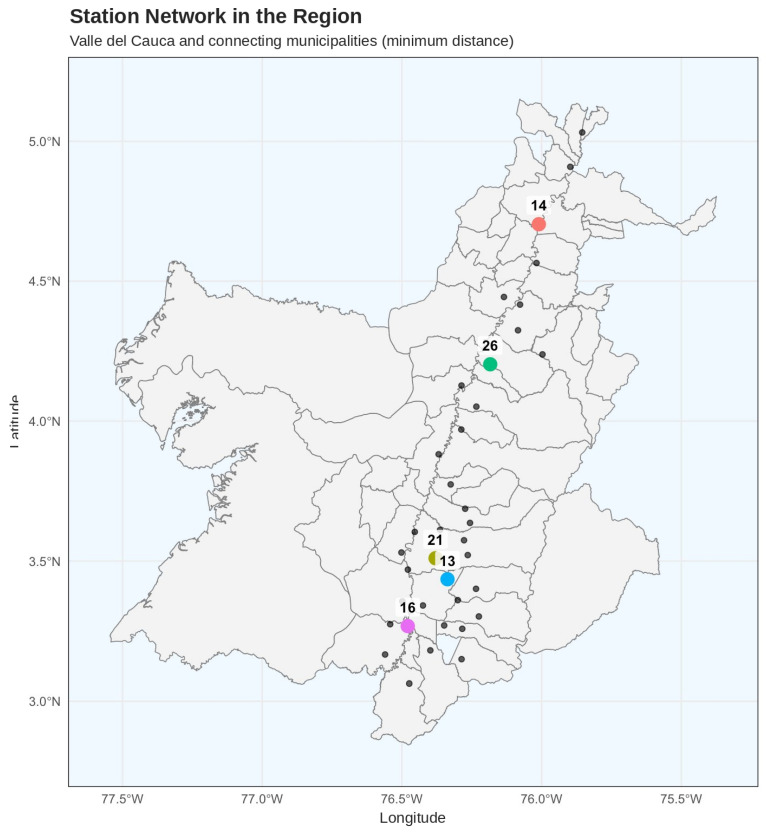
Hydroclimatic station network in the Valle del Cauca region. Colored points represent the five representative stations selected via *k*-means clustering (codes 14, 21, 26, 13, and 16). Small black dots correspond to the full 62-station network.

Ground-based daily measurements were obtained from 62 automatic meteorological stations (EMAs) operated by IDEAM [14] for the period 2016–2025. Five variables were extracted at daily resolution: minimum temperature ($T_{\min}$, °C), maximum temperature ($T_{\max}$, °C), relative humidity (RH, %), solar radiation (Rad, kWh m^−2^ day^−1^), and a binary precipitation occurrence indicator ($P_{\mathrm{bin}}\in \left\{0,1\right\}$; a rain event was defined as a daily total >0.1 mm). To span the main climatic gradients of the department while keeping the analysis tractable, a *k*-means clustering algorithm (k=5) was applied to geographic coordinates, and one station per cluster was selected (codes 14, 21, 26, 13, and 16) based on maximising within-cluster spatial coverage; the silhouette coefficient was used to confirm k=5 as the optimal number of clusters (Figure 1).

Table 1 summarises how the two station sets are used throughout the paper. Model fitting and the experimental grid evaluation use all 62 EMAs; the per-station *ranges* reported in the final performance tables (Table 10, Table 11, Table 12, Table 13 and Table 14) and the spatial LOSO validation are computed on the 37 EMAs that retain complete-case records for all five variables and their covariates over the hold-out period. The five representative stations are used exclusively for visualisation in exploratory figures. Missing data were handled by complete-case analysis within each station–day; station–years with fewer than 200 valid daily observations for a given variable were flagged but retained with available records. The reduction from 62 to 37 stations in the final per-station tables reflects the joint complete-case requirement across all five variables simultaneously (a station missing any single covariate stream in the 2023–2025 window is excluded from the per-station summary, though it still contributes to model fitting); this is a conservative reporting choice and does not affect the fitted models.

### 2.3. Satellite Data and Systematic Biases

**Figure 2:**
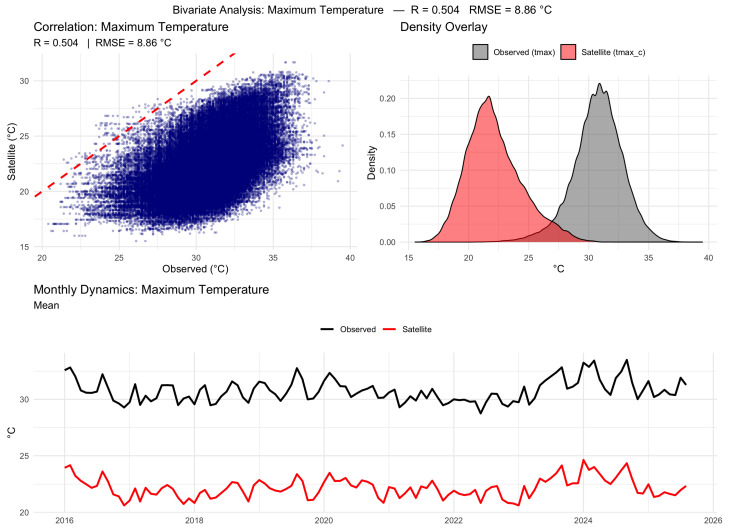
Bivariate analysis of maximum temperature (°C): R=0.504; $\mathrm{RMSE}=8.86^{\;\circ }$C. (**Top left**): scatter plot; the dashed red line is the 1:1 identity line (perfect agreement), *not* a regression fit, so the systematic displacement of the point cloud below it directly visualises the cold bias. (**Top right**): overlaid kernel density estimates of the observed $T_{\max}$ (“Observed”, gray) and the reanalysis $T_{\max}$ (“Satellite”, red); the ≈$8.5^{\;\circ }$C–$9^{\;\circ }$C separation between the two modes is the additive cold bias. (**Bottom**): monthly mean dynamics (black: observed; red: reanalysis), showing the parallel but offset trajectories that confirm the cold bias.

Satellite estimates were obtained from the NASA POWER reanalysis product [15] at a nominal spatial resolution of $0.5^{\circ }$ (≈55 km). A systematic exploratory analysis revealed substantial and physically interpretable biases relative to surface observations. Figure 2 illustrates the cold bias for maximum temperature (R=0.504, $\mathrm{RMSE}=8.86^{\;\circ }$C), where the satellite distribution is entirely shifted to the left relative to the observed distribution, with a nearly constant additive offset of ≈$8.5^{\;\circ }$C–$9^{\;\circ }$C throughout the study period.

**Figure 3:**
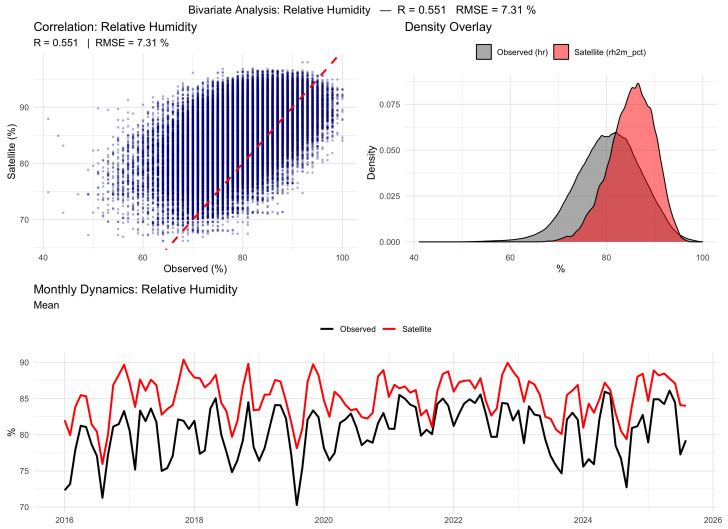
Bivariate analysis of relative humidity (%): R=0.551; RMSE=7.31%. Scatter plot of daily paired satellite–surface values (blue dots); the dashed red line is the 1:1 identity line. The overestimation visible in the scatter plot (point cloud predominantly above the identity line) is consistent with the expected effect of the cold bias operating through the Clausius–Clapeyron relation: a reanalysis product that underestimates temperature tends to overestimate the saturation-relative humidity ratio.

Figure 3 shows the corresponding relative humidity analysis (R=0.551, RMSE=7.31%). The NASA POWER reanalysis overestimates RH by ≈4.5% on average consistent with the expected effect of the cold bias operating through the Clausius–Clapeyron relation and acts as a low-pass filter that suppresses extreme dry days below 66%.

We invoke the Clausius–Clapeyron relation, rather than the ideal-gas law, because the quantity of interest is *relative* humidity. A reanalysis product that underestimates *T* by 5–9 °C therefore underestimates $e_{s}\left(T\right)$ and, for a comparable actual moisture content, overestimates the RH ratio. We present this mechanism as a physically motivated *association* consistent with the data, not as a proven causal pathway (see Section 6 and Section 8).

**Figure 4:**
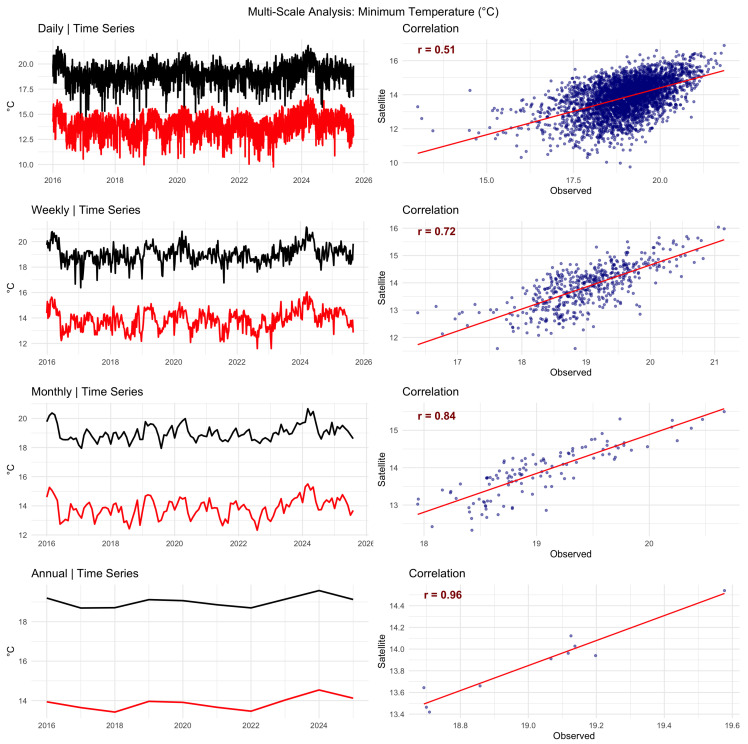
Multi-scale analysis of minimum temperature (°C). Each row corresponds to a temporal aggregation scale (daily, weekly, monthly, and annual), obtained by plain arithmetic averaging of the daily series over each window (no smoothing filter applied). (**Left column**): time series (black: observed; red: satellite). (**Right column**): scatter plot with Pearson coefficient *r*, with the 1:1 identity line shown for reference. The systematic improvement in *r* with aggregation scale (0.51→0.72→0.84→0.96) reflects the averaging-out of independent daily noise: the satellite captures low-frequency variability reliably while showing high daily noise. The per-panel axis ranges differ because the dynamic range of the data shrinks with aggregation; this rescaling is cosmetic and does not affect *r*.

The multi-scale correlation analysis (Figure 4) reveals that satellite–surface agreement improves substantially with temporal aggregation for all variables: for minimum temperature, r=0.51 (daily) → r=0.84 (monthly) → r=0.96 (annual). Here “multi-scale” refers to evaluating the satellite–surface agreement at successive temporal aggregation scales (daily, weekly, monthly, and annual); the aggregated series are plain arithmetic means of the daily values over each window, with *no* smoothing filter or moving-average kernel applied, so the reduction in scatter at coarser scales reflects the averaging-out of independent daily noise rather than any imposed smoothing. The vanishing of small high-frequency fluctuations in the annual panel is the expected consequence of averaging ≈365 daily values per point. This pattern confirms that the satellite captures low-frequency hydroclimatic variability with sufficient fidelity to serve as a functional covariate, and justifies the statistical calibration strategy adopted in this paper.

The key statistics for all five variables are summarized in Table 2, showing the magnitude of the biases that motivate the GMFAMM correction.

## 3. The Generalized Multivariate Functional Additive Mixed Model

This section specifies the Generalized Multivariate Functional Additive Mixed Model (GMFAMM) and its adaptation to our setting. Throughout, RH denotes relative humidity and Rad denotes solar radiation; the multivariate functional principal component analysis (MFPCA) used to represent the shared latent process is introduced in Section 3.4.

### 3.1. Outcome Space and Notation

Let $Y_{i}\left(t\right)={\left(Y_{i}^{\left(1\right)}\left(t\right),\ldots ,Y_{i}^{\left(K\right)}\left(t\right)\right)}^{⊤}$ be the *K*-dimensional daily hydroclimatic response at station *i* on day *t*, partitioned as

(1) $$Y_{i}\left(t\right)={\left(\underset{\mathrm{continuous}}{\underset{︸}{Y_{i}^{\left(1\right)},\ldots ,Y_{i}^{\left(K_{c}\right)}}},\;\underset{\mathrm{binary}}{\underset{︸}{Y_{i}^{\left(K_{c}+1\right)},\ldots ,Y_{i}^{\left(K\right)}}}\right)}^{⊤},$$

where $K_{c}=4$ ($T_{\min}$, $T_{\max}$, RH, Rad) and $K-K_{c}=1$ ($P_{\mathrm{bin}}$). The outcome space is the mixed product $Y=R^{K_{c}}\times {\left\{0,1\right\}}^{K-K_{c}}$.

### 3.2. Model Specification

The GMFAMM [11] specifies, for each variable k∈{1,…,K}, a conditional distribution from the exponential family:

(2) $$Y_{it}^{\left(k\right)}|X_{i},\Lambda_{i}\;∼\;D^{\left(k\right)}\;\left(\theta_{i1}^{\left(k\right)}\left(t\right),\ldots ,\theta_{iR^{\left(k\right)}}^{\left(k\right)}\left(t\right)\right),$$

where $D^{\left(k\right)}$ is variable-specific (N for temperatures, Gamma for radiation, and Bernoulli for precipitation occurrence), and the location parameter is linked to the additive predictor via a canonical link $g_{k}$:

(3) $$g_{k}\;\left(\mu_{i}^{\left(k\right)}\left(t\right)\right)=\underset{\mathrm{fixed}\;\mathrm{additive}\;\mathrm{predictor}}{\underset{︸}{\beta_{0}^{\left(k\right)}\left(t\right)+\sum_{l=1}^{L}f_{l}^{\left(k\right)}\;\left(x_{i}^{\left(l\right)},t\right)+u_{i}^{\left(k\right)}}}+\underset{\mathrm{shared}\;\mathrm{latent}\;\mathrm{process}}{\underset{︸}{\Lambda_{0i}\left(t\right)\;\nu_{0}^{\left(k\right)}}}+\varepsilon_{it}^{\left(k\right)}.$$

In Equation (3), $\beta_{0}^{\left(k\right)}\left(t\right)$ is the functional intercept; the sum $\sum_{l}f_{l}^{\left(k\right)}$ represents flexible covariate effects of the satellite predictors $x_{i}^{\left(l\right)}$ (implemented as either linear terms or smooth P-splines); $u_{i}^{\left(k\right)}=s\left({\mathrm{id}}_{i},\mathrm{bs}=\mathrm{re}\right)$ is the station random intercept; and $\Lambda_{0i}\left(t\right)$ is the shared station-level latent Gaussian process, with loading $\nu_{0}^{\left(k\right)}$ on variable *k* and $\varepsilon_{it}^{\left(k\right)}$ the idiosyncratic error. All additive effects include a cyclic cubic spline for day-of-year seasonality $s({\mathrm{doy}}_{t},\;\mathrm{bs}=\mathrm{cc},\;k=15)$ and a regression spline for the long-term trend s(t,bs=cr,k=15).

### 3.3. Distributional Families and Link Functions

The heterogeneity of the five target variables requires variable-specific distributional assignments:

(4) $$\begin{array}{cc} T_{\min},\;T_{\max}:\; & Y^{\left(k\right)}∼N\left(\mu ,\sigma^{2}\right),\;g\left(\mu \right)=\mu . \end{array}$$

(5) $$\begin{array}{cc} \mathrm{Rad}:\; & Y^{\left(k\right)}∼\mathrm{Gamma}\left(\mu ,\phi \right),\;g\left(\mu \right)=\log\mu . \end{array}$$

(6) $$\begin{array}{cc} P_{\mathrm{bin}}:\; & Y^{\left(k\right)}∼\mathrm{Bernoulli}\left(\pi \right),\;g\left(\pi \right)=\mathrm{logit}\left(\pi \right). \end{array}$$

For relative humidity, the Gaussian and Gamma families are both evaluated (see Section 5). The Gamma and Binomial choices are physically motivated: solar radiation is strictly positive with approximately constant coefficient of variation, and precipitation occurrence is inherently binary.

**Note on Poisson inclusion.** Poisson models for RH and Rad are included in the experimental grid (Table 4) as deliberate reference configurations to quantify the empirical cost of distributional misspecification for continuous variables [16].

### 3.4. Shared Latent Process and MFPCA Representation

The latent process $\Lambda_{0i}\left(t\right)∼\mathrm{GP}\left(0,K_{0}\left(t,\cdot \right)\right)$ is the central innovation of the GMFAMM relative to five independent GAMMs. Its truncated Karhunen–Loève (KL) expansion at order $M_{0}$ is

(7) $$\Lambda_{0i}^{\left(k\right)}\left(t\right)\approx \sum_{m=1}^{M_{0}}\rho_{0im}\;\psi_{0m}^{\left(k\right)}\left(t\right),$$

where the random scores $\rho_{0im}$ are shared across all *K* dimensions for each component *m* the key mechanism through which the model captures cross-variable dependence. The multivariate functional principal components (MFPCs) $\psi_{0m}^{\left(k\right)}\left(t\right)$ are estimated via the two-step procedure of Happ and Greven [17]: separate univariate FPCAs per dimension, followed by an eigenanalysis of the weighted cross-covariance matrix of the univariate scores. In this application, the $M_{0}=1$ component is retained, explaining 38.5% of the total joint residual variation (eigenvalue ${\hat{\nu }}_{01}=1.924$), consistent with the dominant Clausius–Clapeyron energy balance mode documented in Section 6. We stress that Equation (7) and the Happ–Greven construction describe the *framework being approximated*:

### 3.5. Methodological Adaptations for Hydroclimatic Data

Four adaptations were required beyond the original GMFAMM framework [11], whose validation was limited to small clinical datasets.

Variance equalization weighting.

Variables with disparate marginal variances ($\sigma_{T_{\max}}^{2}\approx 4^{\;\circ }C^{2}$ vs. $\sigma_{P_{\mathrm{bin}}}^{2}\approx 0.25$) cause the multivariate eigendecomposition to collapse onto the highest-variance dimension without standardisation. These variances are the empirical (sample) variances of the standardised marginal GAMM residual series computed on the training partition, one per variable; the values quoted are representative magnitudes that illustrate the scale disparity. All marginal residual series are transformed to unit variance before the MFPCA eigenanalysis.

MCMC convergence engineering.

Initial runs with default BAMLSS priors on the variance parameters $\nu_{0m}$ failed to mix for the binary precipitation dimension, where logit-link score updates become numerically unstable when predicted probabilities approach 0 or 1. This was resolved by tightening the inverse-Gamma hyperprior on $\nu_{01}$ (shape =0.001+n/2, rate $=0.001+\rho_{0}^{(m)⊤}\rho_{0}^{\left(m\right)}/2$) and imposing an early stopping criterion on the score magnitude during the first 500 burn-in iterations. Gelman–Rubin $\hat{R}\leq 1.05$ for all monitored parameters confirms convergence.

Station-level satellite calibration.

Prior to model fitting, each satellite covariate $x_{i}^{\left(l\right)}$ undergoes a station-level linear bias correction on the training set: ${\hat{x}}_{it,\mathrm{cal}}^{\left(l\right)}={\hat{\alpha }}_{i}^{\left(l\right)}+{\hat{\gamma }}_{i}^{\left(l\right)}x_{it}^{\left(l\right)}$. This removes the systematic additive and multiplicative biases (in particular the $5^{\;\circ }$C–$9^{\;\circ }$C cold bias documented in Section 2.3). Correction factors are applied verbatim to the test set to ensure strict out-of-sample evaluation. This calibration is station-specific and is therefore used only at gauged locations; for ungauged deployment the regional smooth terms absorb the bias instead, a configuration whose spatial validity is established by the leave-one-station-out analysis of Section 6.6.

Scale of application.

The dataset (n=62 stations, K=5 variables, 10 years of daily records 2016–2025, ≈200,000 station–day observations) is approximately two orders of magnitude larger than the simulation studies in Volkmann et al. [11]. All smoothing parameters are estimated by fast REML using mgcv::bam [18] the bam (“big additive models”) fitting routine of the R package mgcv, designed for generalized additive models on large datasets via a memory-efficient block QR decomposition rather than full Bayesian MCMC, to ensure tractability at this scale.

## 4. Experimental Design and Evaluation Protocol

### 4.1. Study Variables and Distributional Families

The experimental grid covers five target variables spanning the full range of exponential family distributions. Table 3 summarises their identifiers, main satellite predictors, and the distributional families evaluated for each.

### 4.2. Model Configurations and Experimental Grid

The full experimental grid results from crossing three design factors: (i) response variable; (ii) distributional family and effect structure (linear vs. smooth P-splines); and (iii) covariate set. For each variable and admissible family–structure combination, the homologous NASA POWER field is combined with all nested subsets of the candidate auxiliary predictors, yielding covariate sets ranging from a single predictor up to four (continuous variables) or five (precipitation occurrence). This allows for the direct assessment of the marginal predictive value of each additional satellite signal. The complete grid spans 224 distinct model configurations (Table 4).

The general model formulas are as follows:

**Gaussian Smooth** (temperatures, humidity baseline):

(8) $$y_{it}∼N\left(\mu_{it},\sigma^{2}\right),\;\mu_{it}=\sum_{l=1}^{L}s_{l}\;\left(x_{it}^{\left(l\right)}\right)+u_{i}+s\left({\mathrm{doy}}_{t}\right)+s\left(t\right).$$

**Gamma Log-Smooth** (solar radiation):

(9) $$y_{it}∼\mathrm{Ga}\left(\mu_{it},\phi \right),\;\log\mu_{it}=\sum_{l=1}^{L}s_{l}\;\left(x_{it}^{\left(l\right)}\right)+u_{i}+s\left({\mathrm{doy}}_{t}\right)+s\left(t\right).$$

**Binomial Logistic Smooth** (precipitation occurrence):

(10) $$y_{it}∼\mathrm{Bin}\left(1,\pi_{it}\right),\;\mathrm{logit}\left(\pi_{it}\right)=\sum_{l=1}^{L}s_{l}\;\left(x_{it}^{\left(l\right)}\right)+u_{i}+s\left({\mathrm{doy}}_{t}\right)+s\left(t\right).$$

In all cases $s_{l}\left(\cdot \right)$ denotes a smooth P-spline with k=12 bases; linear variants replace $s_{l}$ with $\beta_{l}x$.

Equations (8)–(10) are the marginal (Stage 1) model templates instantiated once for every cell of the experimental grid of Table 4: for a given response variable, the family fixes which of the three templates applies, the effect structure fixes whether each covariate enters through $s_{l}\left(\cdot \right)$ or $\beta_{l}x$, and the covariate set fixes the predictors $\left\{x^{\left(l\right)}\right\}$ entering the sum. Each fitted instance is scored out-of-sample (Section 5); the single best-performing instance per variable becomes the deployed marginal model (Table 6) and the Stage 1 baseline against which the shared latent step of Section 6 is measured. The shared latent term $\Lambda_{0i}\left(t\right)\nu_{0}^{\left(k\right)}$ of Equation (3) is added on top of these marginals in Stage 2.

### 4.3. Temporal Split and Evaluation Metrics

A strict chronological split is used throughout: the first seven years of the available record (2016–2022) constitute the training set and the remaining three years (2023–2025) the hold-out test set, yielding a 70/30 partition by year (approximately 72/28 by observation count after accounting for station availability). This ensures that all reported metrics reflect genuinely prospective predictive skill, analogous to an operational forecasting scenario; random cross-validation would yield optimistically biased estimates for autocorrelated time series [18]. The primary ranking criterion is the RMSE for the four continuous response variables and Log-Loss for the binary precipitation occurrence indicator, with AUC being reported as a secondary diagnostic for $P_{\mathrm{bin}}$.

## 5. Results I: Marginal Model Comparison

### 5.1. Distributional Family and Effect Structure

**Figure 5:**
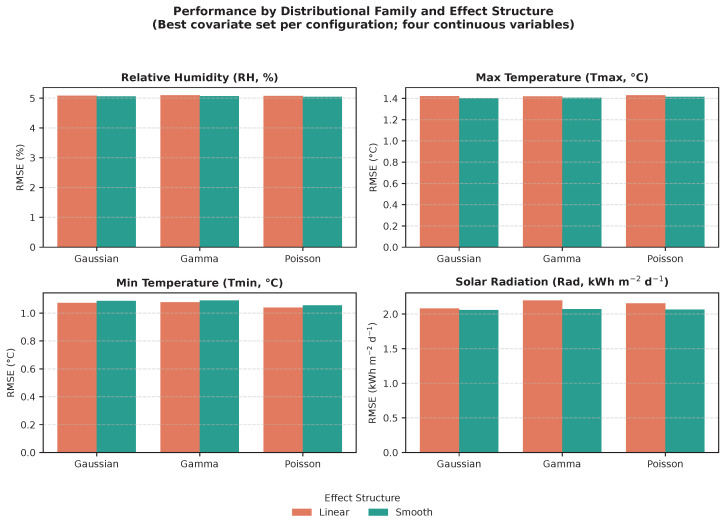
Out-of-sample RMSE by distributional family and effect structure for the four continuous response variables. Each bar represents the best covariate set for the corresponding family–structure combination. Lower values indicate better predictive performance.

Figure 5 provides a visual summary of the best out-of-sample RMSE achieved by each distributional family and effect structure combination across the four continuous response variables (precipitation occurrence is summarised separately in Table 6 and Table 14).

The choice of distributional family has only a small impact once the covariate set is fixed. For the four continuous variables, the three families evaluated, Gaussian, Gamma, and the deliberately misspecified Poisson benchmark, differ by less than about 2% in out-of-sample RMSE (Figure 5), so the covariate set, not the family, is the dominant factor. We adopt the Gaussian family for the temperatures and relative humidity; for relative humidity this choice is confirmed against the natural bounded-support alternatives in Table 5 (Gaussian 5.08% vs. beta 5.14% and logit-normal 5.20%), consistent with RH exhibiting approximately constant variance in the interior of its range rather than the mean-dependent variance those families impose. For solar radiation the Gamma and Gaussian families perform within roughly 0.5% of each other; we retain Gamma on physical grounds (a strictly positive, multiplicatively noisy variable, as specified in Section 3), with the Gaussian marginal used in the joint analysis of Section 6. Poisson is retained in the grid solely as a misspecification benchmark and is not a deployment candidate.

A direct sensitivity analysis confirms the family choice for relative humidity empirically. Refitting the optimal RH specification under the Gaussian, beta, and logit-normal families on the same chronological hold-out (Table 5) shows that the Gaussian achieves the lowest out-of-sample RMSE, with the bounded-support alternatives marginally worse consistent with RH exhibiting approximately constant variance in the interior of its observed range rather than the mean-dependent variance those families impose.

### 5.2. Smooth Versus Linear Effect Structure

**Figure 6:**
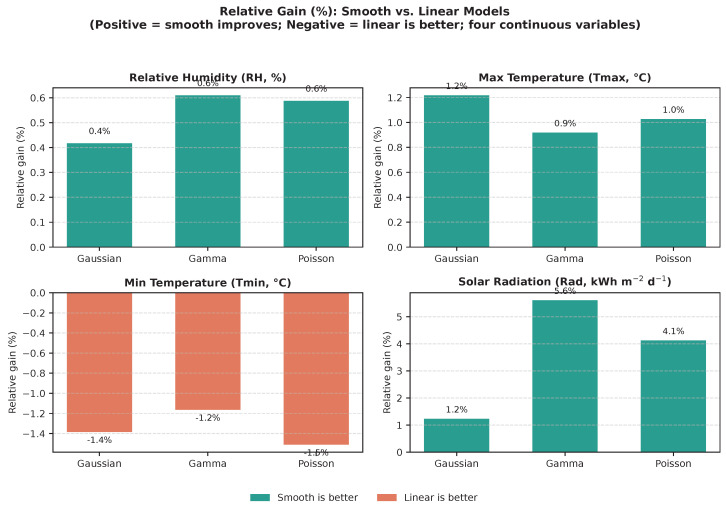
Relative gain (%) of smooth (P-spline) vs. linear effect structure for the best covariate set of each family–response combination. Positive values favor smooth; negative values favor linear.

Figure 6 compares smooth P-spline against linear covariate terms. The differences are modest and variable-specific: smooth terms result in small gains for maximum temperature, relative humidity and solar radiation (approximately 1%, 0.5% and 1–6% respectively), whereas for minimum temperature a linear structure is marginally preferable (about 1.4%). Because these effect–structure differences are small.

### 5.3. Covariate Importance

**Figure 7:**
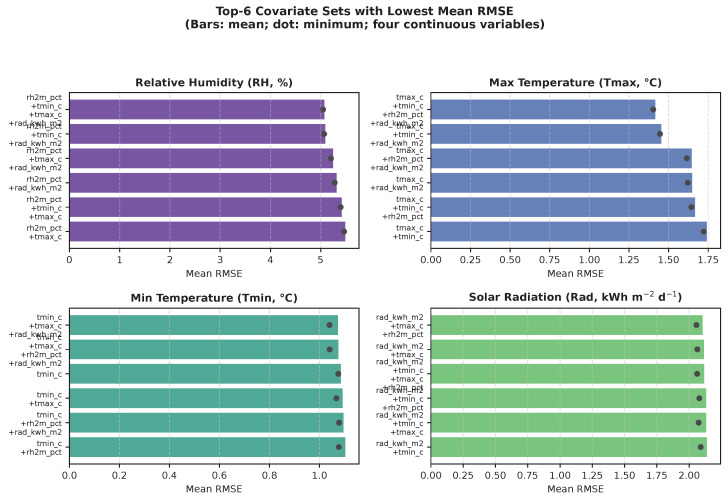
The top six covariate sets ranked by mean out-of-sample RMSE per continuous response variable, averaged across distributional families and effect structures. Bars: mean score; dot: minimum score achieved by the best family–structure combination.

Figure 7 displays the six covariate sets with the lowest mean RMSE for each continuous response variable, averaged over all admissible family–structure combinations. A clear hierarchy of satellite predictors emerges. For temperature variables, the homologous NASA POWER field alone provides a strong baseline; the most informative additions are solar radiation (ALLSKY_SFC_SW_DWN) for maximum temperature and relative humidity (RH2M) for minimum temperature. For relative humidity, the best-performing sets invariably include both T2M_MIN and ALLSKY_SFC_SW_DWN, consistent with the Clausius–Clapeyron thermodynamic constraint. For precipitation occurrence (Table 6), RH2M is the most informative additional predictor.

### 5.4. Deployed Marginal Configurations

Table 6 reports the configuration deployed for each response variable. These were chosen to combine strong out-of-sample performance with parsimony and physical interpretability rather than to minimise RMSE at any cost: as Figure 7 shows, adding further covariates yields only marginal additional reductions, which we trade off against overfitting risk and operational simplicity. These deployed configurations serve as the Stage 1 baseline for the joint modelling analysis of Section 6.

The best temperature models achieve RMSE values of $1.16^{\;\circ }$C and $1.63^{\;\circ }$C for $T_{\min}$ and $T_{\max}$ respectively, representing approximately 80% and 82% improvements over the unadjusted reanalysis biases of $5.67^{\;\circ }$C and $8.86^{\;\circ }$C documented in Table 2. For relative humidity, the best model reduces RMSE from the raw reanalysis baseline to 5.08%, an approximately 30% relative improvement. For precipitation occurrence, AUC =0.795 indicates moderate-to-good discriminative ability.

The RMSE values reported in this table are used directly as the Stage 1 baseline for the joint modelling analysis of Section 6; all subsequent comparisons are made against this correctly specified marginal model, so that the reported gains of the shared latent step are measured on a like-for-like basis.

## 6. Results II: Two-Stage Joint Correction Versus Independent GAMMs

### 6.1. Cross-Variable Residual Correlations

**Figure 8:**
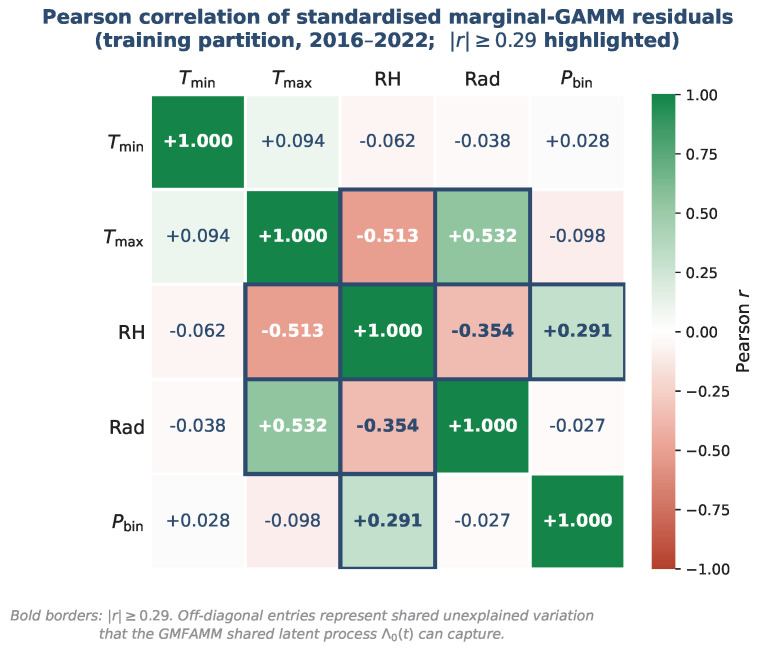
Pearson correlation matrix of standardised marginal GAMM residuals (**training partition, 2016–2022**), for all five variables including precipitation occurrence ($P_{\mathrm{bin}}$). Cells with |r|≥0.29 are outlined in bold; these are the pairs from which the joint models draw their cross-variable information. Off-diagonal entries represent shared unexplained variation that the GMFAMM shared latent process $\Lambda_{0}\left(t\right)$ can capture. Values reported in the narrative ($r_{T_{\max}\text{-}\mathrm{Rad}}=+0.532$, $r_{T_{\max}\text{-}\mathrm{RH}}=-0.513$, $r_{\mathrm{Rad}\text{-}\mathrm{RH}}=-0.354$, $r_{\mathrm{RH}\text{-}P_{\mathrm{bin}}}=+0.291$) correspond to this training partition; the equivalent strongest hold-out values are $r_{T_{\max}\text{-}\mathrm{RH}}=-0.41$ and $r_{T_{\max}\text{-}\mathrm{Rad}}=+0.544$ (see Appendix B).

A central claim of the GMFAMM framework is that jointly modelling the *K* hydroclimatic variables through a shared latent process $\Lambda_{0i}\left(t\right)$ improves predictive performance beyond what independent marginal GAMMs achieve. To test this claim, we first examine the Pearson correlation matrix of all five sets of standardised marginal residuals and the four continuous variables together with the Bernoulli Pearson residual of $P_{\mathrm{bin}}$ on the *training* partition (Figure 8). These off-diagonal dependences are precisely the shared unexplained variation that the latent process of Section 6.2 is designed to capture across the *K* outcomes.

Four off-diagonal correlations exceed |r|=0.29 on the training partition, which are all physically interpretable within the thermodynamic framework of the Andean tropical climate:

$T_{\max}$–Rad: r=+0.532. When the satellite solar radiation field under-predicts (positive residual), surface $T_{\max}$ also tends to be under-predicted by its marginal model consistent with the direct control of incoming shortwave radiation on peak diurnal warming.

$T_{\max}$–RH: r=−0.513. The negative sign is consistent with the Clausius–Clapeyron thermodynamic framework: days on which $T_{\max}$ is anomalously warm tend to produce anomalously low relative humidity, and both marginal models fail to capture the coupling simultaneously.

Rad–RH: r=−0.354. Cloud cover attenuates incoming shortwave radiation and increases surface humidity; partial cloud effects not resolved by the NASA POWER $0.5^{\;\circ }$ (≈55 km) grid leave correlated residuals in both variables.

RH–$P_{\mathrm{bin}}$: r=+0.291. Days on which the marginal model under-predicts relative humidity (positive RH residual) tend to coincide with under-predicted rain occurrence, consistent with elevated near-surface moisture favouring precipitation; this is the only cross-variable coupling involving the binary outcome that exceeds the outlining threshold, and it motivates retaining $P_{\mathrm{bin}}$ in the shared latent representation even though its joint predictive gain is treated as exploratory (Section 6.3).

By contrast, $T_{\min}$ residuals show near-zero correlation with all other variables (|r|≤0.10), confirming that the nocturnal cooling process is largely independent of the daytime energy balance captured by the remaining variables.

### 6.2. Shared Latent Process: PCA Analysis

**Figure 9:**
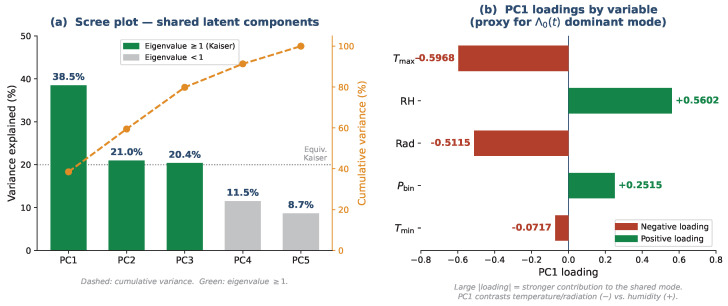
(**a**) Scree plot of the PCA on the matrix of standardised marginal residuals. PC1 (eigenvalue = 1.924, explaining 38.5% of total joint residual variation) is the operational proxy for the dominant GMFAMM latent process $\Lambda_{0}\left(t\right)$. PC2 and PC3 also exceed the Kaiser criterion (eigenvalue >1). Dashed line: cumulative variance. (**b**) PC1 loadings by variable. The dominant mode contrasts solar radiation and $T_{\max}$ (negative loadings) against relative humidity (positive loading), reflecting the Clausius–Clapeyron-driven thermodynamic coupling of the inner-Andean climate.

Figure 9 presents the scree plot and PC1 loadings from the PCA of the five-variable residual matrix. PC1 has eigenvalue 1.924>1 (Kaiser criterion) and accounts for 38.5% of the total joint residual variation. Under a $M_{0}=1$ GMFAMM specification, this component corresponds to the single MFPC ${\hat{\psi }}_{01}\left(t\right)$ and its associated random scores $\rho_{0i1}$, pooling information across all *K* outcomes. This five-variable decomposition is *descriptive*, used here to characterise and physically interpret the dominant shared mode (its loadings appear in Figure 9b); the principal component actually *added to the predictor* of each recipient variable in the joint correction of Section 6.3 is instead computed leave-one-variable-out, excluding that recipient’s own residual, so that no variable is ever corrected using its own observation.

We retain $M_{0}=1$ on the basis of three complementary criteria, and we are explicit about the trade-off involved. First, while PC2 (eigenvalue =1.049) and PC3 (eigenvalue =1.019) also exceed the Kaiser threshold, both lie within 5% of the boundary value of 1.0, placing them in the acknowledged ambiguity zone of that criterion [19]. Second, and more importantly, the PC1 loadings display a clear and physically interpretable structure $T_{\max}$, Rad negative, and RH positive directly consistent with the Clausius–Clapeyron energy balance mode of the Valle del Cauca climate system. The PC2 and PC3 loadings do not correspond to a recognisable atmospheric process in this context, suggesting they capture residual noise rather than a coherent latent signal. Third, under the MFPCA framework of Happ and Greven [17], retaining components with marginal eigenvalues risks overfitting the shared latent structure, particularly in a dataset with heterogeneous response types.

We stress that this is a deliberate choice and not a claim that additional components are useless: the sensitivity analysis of Table 7 shows that adding PC2 and PC3 yields further out-of-sample reductions that are small for $T_{\min}$, $T_{\max}$ and RH but *non-trivial for Rad* (from −9.4% at $M_{0}=1$ to −15.4% at $M_{0}=3$). The apparent tension with the RMSE-driven model selection of Section 5 is resolved by noting that the two selections answer different questions. The marginal grid search selects the *deployed* per-variable specification by pure out-of-sample RMSE, because that model is evaluated at *every* queried coordinate. The choice of $M_{0}$, by contrast, governs only the *conditional* shared latent term, which is applied solely at gauged points within 2 km of an EMA (Section 6.3), a small fraction of the operational queries. There, we prioritise a single, physically interpretable and plausibly transferable mode over extracting marginal RMSE from components that sit in the Kaiser ambiguity zone, lack a recognisable atmospheric structure, and whose gains may partly reflect hold-out-specific structure rather than a transferable latent signal. Reframed in transferability terms, PC1 is the only component whose loading structure is physically coherent and that we therefore expect to generalise across the network, whereas the additional out-of-sample reduction from PC2/PC3 may not survive a spatial re-derivation of the components and is, in that sense, not yet established as transferable. For solar radiation specifically, $M_{0}=3$ yields a substantially larger conditional gain (−15.4% vs. −9.4% at $M_{0}=1$); accordingly, *at gauged points where minimising RMSE is the priority, $M_{0}=3$ is the recommended setting for Rad*, whereas $M_{0}=1$ is retained as the conservative default for ungauged deployment on the grounds of interpretability and expected spatial transferability. These additional components lack a clear physical interpretation and are not retained in the *default* deployed model, but because the gain is non-trivial we report the full $M_{0}\in \left\{1,2,3\right\}$ results (Table 7) and expose $M_{0}$ as a configurable option in the deployed application, so that this trade-off is left to the user rather than imposed. A definitive test of whether the PC2/PC3 gains are genuinely transferable rather than hold-out-specific requires re-deriving the *shared components* themselves under a spatial-block/leave-one-station-out design. We note that the spatio-temporal block validation of Section 6.6 already establishes that the *marginal* regional correction transfers across space on the difficult 2023–2025 window; what remains for future work is the analogous re-derivation of the shared latent components ($M_{0}>1$), which we leave to the planned follow-up (Section 8).

The PC1 loadings confirm the expected physical signs: $\nu_{0}^{T_{\max}}=-0.597$, $\nu_{0}^{\mathrm{RH}}=+0.560$, $\nu_{0}^{\mathrm{Rad}}=-0.512$, and $\nu_{0}^{T_{\min}}=-0.072$. The dominant mode thus opposes two groups of variables: when the observed values exceed the predictions of $T_{\max}$ and Rad (positive residuals), the RH residual is negative consistent with the Clausius–Clapeyron thermodynamic framework. The near-zero loading of $T_{\min}$ (−0.072) confirms that nocturnal minimum temperature is largely decoupled from the dominant daytime energy balance mode. Table 8 reports the full eigenvalue decomposition.

### 6.3. Predictive Gain of Joint Modelling

To quantify the contribution of the shared latent process, we implement a two-stage approximation to the full simultaneous GMFAMM. Stage 1 fits the optimal marginal GAMM for each variable independently (Section 5). Stage 2A (${\mathrm{Joint}}_{\mathrm{cross}}$) appends the standardised residual of a donor variable $k^{\prime }$ to the additive predictor of a recipient variable *k* as an additional smooth effect $s\;\left({\hat{\varepsilon }}^{\left(k^{\prime }\right)},\;k=8\right)$. Stage 2B (${\mathrm{Joint}}_{\mathrm{PC}1}$) uses the first principal component of the marginal residual matrix as a scalar proxy for the dominant GMFAMM latent process $\Lambda_{0}\left(t\right)$.

**Absence of information leakage.** We emphasise that donor residuals in the hold-out period (2023–2025) are obtained exclusively via predict() applied to the Stage 1 model trained on 2016–2022 data: ${\hat{\varepsilon }}_{t,\mathrm{test}}^{\left(k^{\prime }\right)}=y_{t,\mathrm{test}}^{\left(k^{\prime }\right)}-{\hat{\mu }}^{\left(k^{\prime }\right)}\left(x_{t,\mathrm{test}};\;{\hat{\theta }}_{\mathrm{train}}\right)$. No future information and no information from the *recipient* variable’s own test-period outcome enters the predictor, so the temporal out-of-sample evaluation is strict, and this holds for *both* joint variants by construction.

This two-stage procedure does not replicate the full GMFAMM, in which all *K* variables and the shared latent scores $\rho_{0im}$ are estimated simultaneously; it is an approximation that isolates the predictive value of the dominant shared mode while keeping estimation tractable at the scale of this dataset. We do *not* claim that the two-stage gains.

Table 9 consolidates the numerical results for the four continuous variables and both joint approaches, evaluated on the chronological 70/30 hold-out against the well-specified marginal baseline of Section 5.

### 6.4. Interpretation and Operational Scope of the Joint Gains

Two conclusions follow from Table 9. First, once the marginal model is correctly specified, the additional value of the shared latent step is *modest and variable-specific*: the dominant Clausius–Clapeyron mode delivers a meaningful conditional reduction for $T_{\max}$ (≈17%, i.e., −16.6%, via PC1), relative humidity (≈10%) and solar radiation (≈9%), and a negligible reduction for $T_{\min}$. PC1 (the shared latent proxy) is the through-line for these gains, although for RH the single-donor ${\mathrm{Joint}}_{\mathrm{cross}}$ variant is marginally better than PC1 (−10.8% vs. −9.8%); both are conditional and of comparable magnitude. This is consistent with the residual correlation structure of Section 6.1, in which $T_{\min}$ is nearly decoupled from the daytime energy balance. Second, the bulk of the bias removal is achieved upstream, by station-level calibration and the marginal GAMM (Section 5 and Table 10, Table 11, Table 12 and Table 13); the joint step refines rather than dominates the correction.

We emphasise an operational consequence that bears on how these gains should be read: because every shared latent gain requires co-located ground observations of the donor variables, the ≈17/10/9% reductions are realised *only* at gauged points (within 2 km of an EMA), and are *not* available at the genuinely ungauged coordinates that constitute the primary use case of *ColClim*. The contribution that does generalise to the operational setting is therefore the marginal regional correction, whose spatial transfer we validate directly in Section 6.6. The joint analysis is best understood as quantifying how much exploitable cross-variable signal remains in the marginal residuals as a methodological result rather than as the headline deployable gain.

We deliberately refrain from asserting that these two-stage gains lower-bound those of the full simultaneous GMFAMM. While simultaneous estimation could in principle recover additional shared signal, better in-sample flexibility does not guarantee lower out-of-sample error, and we have no empirical estimate of the full model at this scale. The honest statement is that the two-stage approximation quantifies the exploitable cross-variable signal remaining in the marginal residuals, and that this signal is concentrated in the $T_{\max}$–RH–Rad energy balance triad. Whether the full GMFAMM materially exceeds these figures is an open, empirical question that we leave to future work (Section 8), as computational constraints at the scale of ≈200,000 station–day observations precluded full simultaneous estimation within the submission timeline.

### 6.5. Deployed Model Final Performance

Table 10, Table 11, Table 12, Table 13 and Table 14 report the performance of the deployed two-stage correction for each of the five variables on the held-out chronological test partition. The “Corrected (after)” column is the deployed pipeline (regional GAMM correction, plus the conditional shared latent term where donor observations are available); the incremental contribution attributable specifically to the shared latent term is isolated on a like-for-like basis in Table 9 and is modest and conditional. The ranges in each table reflect variability across the 37 EMAs with complete-case hold-out records (the models are fitted on all 62); inter-station variability was assessed by computing per-station metrics and reporting the 10th–90th percentile range across stations. A non-parametric bootstrap (B=500 resamples) was used to verify that the differences between the “before” and “after” columns are statistically significant (p<0.01) for all metrics and all five variables; the full bootstrap confidence intervals are reproducible from the openly available evaluation scripts (see Data Availability).

Two distinct baselines must not be conflated when reading these tables. The per-station tables (Table 10, Table 11, Table 12 and Table 13) compare against a *station-specific* linear calibration, which is itself strong at gauged sites; the shared latent gains of Table 9, by contrast, are measured *pooled*, on the donor-complete subset (n=36,727), against the *regional* marginal GAMM. The ≈17% $T_{\max}$ reduction in Table 9 is therefore not directly readable from the narrow “linear-only → corrected” gap in Table 11: at a gauged station, the station-specific linear step already captures most of the additive offset that the regional GAMM and the shared latent term recover pooled. The two views are complementary rather than contradictory: the per-station tables report deployed accuracy at instrumented sites, whereas Table 9 isolates the incremental cross-variable signal on a like-for-like basis.

Three patterns stand out from the three-stage performance decomposition. First, station-level linear calibration alone removes the bulk of the systematic bias for temperature variables (≈80–83% median RMSE reduction for $T_{\min}$ and $T_{\max}$), confirming that the dominant error is an additive cold offset consistent with the findings of Section 2.3. Second, beyond the linear baseline, the regional GAMM correction yields a further reduction for temperatures, and a larger relative gain for relative humidity, whose bias is driven by cross-variable Clausius–Clapeyron coupling rather than a simple additive offset in RH itself. The contribution attributable specifically to the shared latent term, measured on a like-for-like basis (Table 9), is modest and conditional (≈17% for $T_{\max}$, ≈10% for RH, ≈9% for Rad, negligible for $T_{\min}$); it complements rather than dominates the marginal correction. Third, for solar radiation, both the linear calibration and the GAMM reduce the raw error (the GAMM giving a further reduction), although Rad remains the hardest variable to correct at a daily resolution (RMSE ≈2 kWh m^−2^ day^−1^; Table 13). For $T_{\min}$, the station-level linear calibration is already near-optimal and the additional GAMM terms do not improve upon it consistent with $T_{\min}$’s decoupling from the daytime energy balance so the deployed $T_{\min}$ correction reduces essentially to the calibration step. Taken together, the three-stage decomposition delimits precisely where the GAMM machinery earns its place over a simple station-level linear correction. For the temperatures, the linear calibration does almost all of the work and the regional GAMM adds little at gauged stations; the GAMM’s decisive contributions are (i) the correction of relative humidity, whose bias is cross-variable rather than a simple additive offset and which the linear step leaves largely intact, and (ii) generalisation to *ungauged* points, where a station-specific linear calibration is by definition unavailable and the regional smooth-plus-random-effect structure is the only correction that transfers (validated in Section 6.6). The elaborate marginal apparatus is thus justified by the operational requirement (arbitrary user coordinates) and by RH, not by marginal temperature gains at gauged sites. The correction removes most of the systematic bias for all five variables: the large raw per-station offsets (up to ≈$-7^{\;\circ }$C for $T_{\min}$, ≈$-10^{\;\circ }$C for $T_{\max}$ and ≈+9% for RH; Table 10, Table 11, Table 12 and Table 13) shrink to a small residual offset and every before–after difference is statistically significant (bootstrap p<0.01 for all pairwise differences). The residual mean bias of the deployed correction stays within ±0.5 units for solar radiation; for the temperatures and relative humidity a small offset remains at individual stations (10th–90th percentile up to ≈$-1.1^{\;\circ }$C for $T_{\max}$ and ≈+2.9% for RH), reflecting that the deployed pipeline applies the *regional* smooth-plus-random-effect correction rather than a station-specific calibration of the mean with the same trade-off, favouring spatial transferability over a perfectly centred per-station fit that underlies the ungauged point design validated in Section 6.6. For precipitation occurrence, the Binomial classifier attains the per-station performance reported in Table 14; we note that the cross-variable RH predictor was *not* part of the reconciled like-for-like out-of-sample analysis of Section 6.3, so any joint improvement for $P_{\mathrm{bin}}$ should be regarded as exploratory pending the same evaluation applied to the continuous variables.

### 6.6. Spatial Generalisation: Leave-One-Station-Out Validation

The hold-out of Section 5.1, Section 5.2, Section 5.3, Section 5.4, Section 6.1, Section 6.2 and Section 6.3 is *temporal*: it tests prediction in unseen years at gauged stations. Because *ColClim* serves user-selected coordinates that may have no nearby station, we additionally assess *spatial* generalisation through leave-one-station-out (LOSO) cross-validation in two designs. In both, for each held-out station the marginal model is refitted on the remaining stations and used to predict the held-out station with the station random intercept excluded (s(id) set to zero) and **without any station-specific linear calibration** i.e., using only information available at a genuinely ungauged point; we never reuse the held-out station’s parameters, which would reintroduce exactly the leakage flagged in the review. The first design (*all years*) trains on the remaining stations’ full record and predicts the held-out station across all ten years, isolating pure spatial transfer. The second design (*2023–2025 block*) trains on the remaining stations’ 2016–2022 record and predicts the held-out station over the difficult 2023–2025 window only; it therefore withholds both the station *and* the future years simultaneously, isolating spatial transfer on exactly the period used by the temporal hold-out a genuine spatio-temporal block.

Table 15 reports both LOSO designs alongside the temporal hold-out. In the all-years design the median LOSO RMSE is $0.96^{\;\circ }$C for $T_{\min}$, $1.25^{\;\circ }$C for $T_{\max}$, and 3.90% for RH each at or below the temporal hold-out, confirming that pure spatial interpolation within the network is comparatively easy and that the dominant source of out-of-sample error is temporal (future-year) extrapolation rather than spatial transfer. The decisive test, however, is the 2023–2025 block, which removes both the station and the difficult window at once and is therefore directly comparable, on the *same* period, to the gauged *Temporal (37)* column. Here the ungauged spatial transfer error is $1.19^{\;\circ }$C for $T_{\min}$, $1.57^{\;\circ }$C for $T_{\max}$, and 4.55% for RH essentially equal to the gauged temporal error for $T_{\min}$ (1.19 vs. 1.16) and *below* it for $T_{\max}$ (1.57 vs. 1.63) and RH (4.55 vs. 5.08). In other words, predicting a previously unseen station over the hard 2023–2025 window, using only regional information, is as accurate as predicting a gauged station over the same window: the regional fixed-effect structure (smooth functions of the raw satellite covariates plus seasonal and trend terms) transfers across space without material loss because the NASA POWER bias in this region is spatially coherent. This earlier “planned” spatio-temporal design is thus now executed, and it provides direct, leakage-free evidence supporting the use of *ColClim* at ungauged points. We retain two honest caveats. First, relative humidity carries a long upper tail in the block design (P90 =8.46%): a minority of stations transfer poorly, consistent with RH being the variable whose bias is cross-variable rather than a simple offset, and accuracy at such locations should not be over-stated. Second, the comparison is not an artefact of the smaller station set recomputing the temporal hold-out on the same complete-case stations leaves it essentially unchanged (1.16, 1.63, 5.08; Table 15). Solar radiation and precipitation occurrence were excluded from this spatial comparison for principled reasons rather than convenience: Rad carries the largest residual error of the five variables and depends strongly on sub-grid cloud processes that the reanalysis does not resolve, making its spatial interpolation the least reliable and warranting a dedicated treatment; $P_{\mathrm{bin}}$ is a binary outcome whose spatial transfer is properly assessed with classifier-specific, distance-stratified metrics rather than RMSE. A full distance-stratified error model and spatial-block cross-validation extended to all five variables remain the methodological focuses of a follow-up paper (Section 8).

## 7. Operational Deployment and Application Limits: The *ColClim* Web Application

### 7.1. Overview and Purpose

We present *ColClim* as one of the paper’s three stated contributions: the operational deployment that turns the validated correction pipeline into a reproducible, openly accessible artefact. The description below is deliberately functional rather than promotional: its purpose is reproducibility (every step from API query to corrected output is documented, and the evaluation and figure-generation scripts are openly available; see Data Availability) and to make explicit the operational scope and the ungauged point caveats established by the validation of Section 6.6. This section has been condensed in revision to its essential architecture, execution flow, and operational limitations.

*ColClim* is an R Shiny web application that makes the GMFAMM bias correction pipeline accessible to practitioners without programming expertise. A live, publicly accessible instance is hosted at https://4d4fmw-david-arango.shinyapps.io/ColClim/ (accessed on 6 June 2026). Its core function is as follows: a user selects a point on an interactive map, specifies a date range and a climatic variable, and the application queries the NASA POWER API for that location, applies the trained GMFAMM correction model, and displays the corrected time series alongside the raw satellite signal. When the selected point falls within 2 km of a physical EMA station, the observed measurements are shown on the same plot as a third reference series, enabling direct quality assessment.

The application is designed around two architectural principles. First, model training is entirely offline: the GMFAMM artefacts (.rds files) are built once on the full historical record, serialized, and loaded at startup in under one second. There is no on-demand retraining, which guarantees that all users receive predictions from the same validated model. Second, the raw NASA POWER signal is always shown alongside the correction, not replaced by it, reflecting a design philosophy of transparency: practitioners should understand the gap between satellite estimates and ground station measurements.

**Correction at user-selected points.** A key operational question is how the station-specific quantities are obtained for an arbitrary coordinate that has no ground station. *ColClim* does *not* require a station-specific ${\hat{\alpha }}_{i},{\hat{\gamma }}_{i}$ at the query point. The deployed correction is the *regional* marginal GAMM: the bias is absorbed by the smooth functions of the satellite covariates together with the seasonal and trend terms, and the station random intercept is set to its population mean (excluded) for ungauged queries. This is precisely the configuration validated out-of-sample in Section 6.6, where it generalised to held-out stations without material loss of accuracy. The station-level calibration and the conditional shared latent term (Section 6.3) are applied only when the query falls within 2 km of an EMA, i.e., where the observed donor series is available; otherwise, the marginal regional correction is returned. The application therefore generalises to ungauged points by design, with the explicit caveat that accuracy is expected to be highest within the convex hull of the training network.

The current version of *ColClim* does not display an explicit uncertainty warning when the queried coordinate is far from the nearest training station. Adding a distance-to-nearest-station chip in the sidebar is the highest-priority interface improvement planned for the next release. Until that indicator is available, users are advised that **predictions for locations well beyond the spatial range spanned by the training network provisionally, more than ∼20 km from the nearest EMA, a threshold to be refined by the planned distance-stratified analysis, should be interpreted as conditional bias-corrected estimates, not as locally validated observations**; the reliability of the correction decreases with distance from the training network, and no spatial uncertainty quantification is currently provided.

### 7.2. Application Architecture

The application has five tabs organized in a fixed side panel plus main content area layout (Table 16). The side panel provides the following: a study zone selector (filtering observed stations by Colombian department); a variable card grid for selecting among the five climatic variables; a reactive model information panel; a date range picker; and a query button that triggers the NASA POWER API call and GMFAMM prediction.

### 7.3. Execution Flow

**Figure 10:**
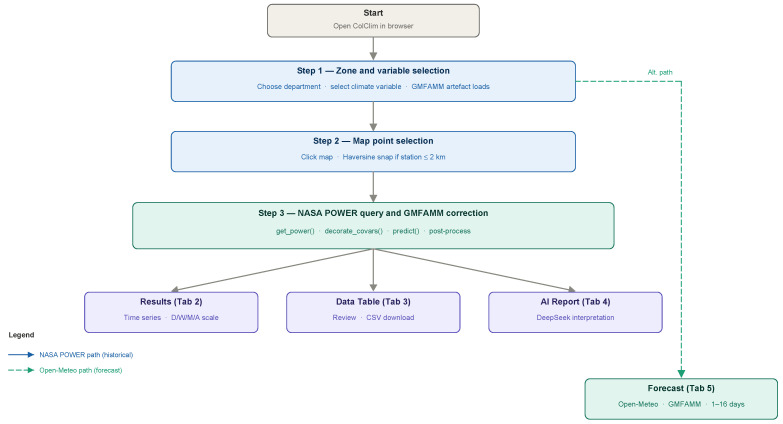
Complete workflow for using *ColClim*. The solid arrow path (Steps 1–3 and Result tabs) corresponds to the historical NASA POWER query pipeline. The dashed arrow path (Tab 5) shows the alternative forecast route via Open-Meteo, which bypasses the NASA POWER query and produces bias-corrected short-range predictions using the same GMFAMM artefacts.

Figure 10 summarises the complete user journey through *ColClim*. The diagram highlights the two data-source branches: the NASA POWER path (solid arrows) for historical bias-corrected analysis, and the Open-Meteo path (dashed arrows) for short-range forecasting, both converging on the same GMFAMM correction artefacts.

A complete historical query for one variable and one year executes in 5–8 seconds from click to plot; the bottleneck is the NASA POWER API call. The automatic station snapping feature uses the Haversine formula; if the nearest station is within 2 km, the selection is adjusted and the observed series is overlaid on the Results tab for direct comparison.

The interface itself is illustrated by screenshots of the map, results, and forecast tabs, which we relegate to Appendix C to keep the main text focused on the methodology and operational scope rather than user interface detail.

### 7.4. Forecast Module

**Figure A7:**
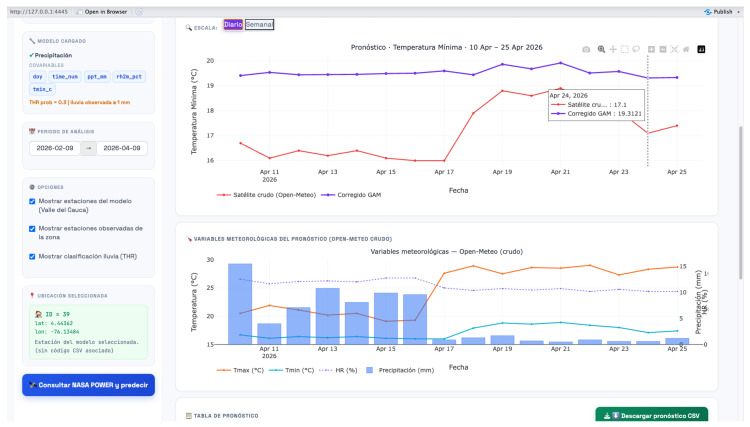
Live application Tab 5 (Forecast) for minimum temperature at Station ID = 39 (lat: 4.44362, lon: −76.13484), forecast period 10–25 April 2026 (16 days). (**Top panel**): raw Open-Meteo signal (red, “Satélite crudo”) vs. bias-corrected forecast (violet, “Corregido GAM”). (**Bottom panel**): multivariate Open-Meteo covariates used as model inputs.

The forecast module (Tab 5) replaces NASA POWER with Open-Meteo [20], a free NWP API providing daily forecasts up to 16 days ahead for any geographic coordinate worldwide. The five-step execution sequence is as follows: (1) fetch NWP frame (fetch_openmeteo_forecast()); (2) decorate with covariates (decorate_covars()); (3) apply a linear calibration step for precipitation to compensate for the systematic scale difference between NASA POWER and Open-Meteo; (4) apply predict(artefact$model, newdata) to obtain $\hat{y}$ on the response scale; and (5) apply physical post-processing (RH clamped to [0,100], radiation unit conversion). A live 16-day minimum temperature forecast is shown in Appendix C (Figure A7).

### 7.5. AI Report Module

Tab 4 provides an interpretive analysis generated by a large language model from the numerical summary statistics of the active query. The report covers the following: statistical interpretation (bias magnitude, RMSE before and after correction); temporal patterns (seasonal variation, ENSO signatures); and the quality and limitations of NASA POWER in the tropical Andean region. The AI module analyses exclusively text and numerical tables; no charts or map images are transmitted. Users should treat the AI report as a first-pass interpretation to be complemented with visual inspection of the Results tab.

## 8. Discussion

This paper proposes and empirically evaluates a two-stage approximation to a GMFAMM for the joint bias correction of five NASA POWER hydroclimatic variables in the Valle del Cauca region of Colombia, and documents its deployment in the open-access *ColClim* web application. The key contributions are threefold. Throughout, the contribution is empirical and operational rather than being a new estimation method: the GMFAMM provides the organising framework for the joint step, but we neither fit nor claim a methodological advance in the full simultaneous model.

First, the systematic evaluation of more than 200 marginal model configurations establishes clear and physically interpretable recommendations for distributional family and covariate selection: Gaussian with smooth P-splines for temperatures and humidity, Gamma for solar radiation, and Binomial for precipitation occurrence. Solar radiation emerges as a consistently informative cross-variable predictor for temperature, while relative humidity serves the same role for precipitation occurrence both consistent with the thermodynamic mechanisms of the Andean climate.

Second, once the marginal model is correctly specified, a two-stage approximation to the GMFAMM shared latent process delivers *modest, conditional* predictive gains for the variables coupled through the surface energy balance: using the first principal component of the marginal residual matrix as a proxy for $\Lambda_{0}\left(t\right)$, th eout-of-sample RMSE falls by ≈17% for $T_{\max}$, ≈10% for RH and ≈9% for solar radiation, with negligible benefit for $T_{\min}$. We are deliberately careful about how these figures are framed. They are conditional on co-located ground observations of the donor variables and are therefore realised only at gauged points; at the ungauged coordinates that constitute *ColClim*’s primary use case they do not apply, and the contribution that generalises operationally is the marginal regional correction (validated spatially in Section 6.6). The bulk of the bias removal (≈80–82% of the raw temperature RMSE) is achieved by station-level calibration and the marginal GAMM, and the joint analysis is best read as a methodological result and as a quantification of the exploitable cross-variable signal remaining in the marginal residuals rather than as the headline deployable gain.. We deliberately do not claim that the two-stage figures lower-bound those of an unfitted full simultaneous GMFAMM. The physical interpretation of the dominant shared mode opposing solar forcing and atmospheric moisture, with PC1 eigenvalue 1.924 and loadings that contrast $T_{\max}$/Rad against RH, is consistent with Clausius–Clapeyron thermodynamic coupling, although we stress that a residual correlation is suggestive, not a proof of causation: definitive attribution would require auxiliary variables such as specific humidity, dew-point temperature, vapour pressure, or cloud cover, which we identify as future work.

Third, the *ColClim* application democratises access to bias-corrected hydroclimatic data in Colombia. The browser-based interface makes the GMFAMM pipeline accessible to agronomists, hydrologists, and public health analysts who need site-specific climate estimates but lack the statistical or programming expertise to generate them from raw satellite products.

Taken together, these contributions should be read against a deliberately honest accounting of where the value resides. Because the shared latent gains are conditional and realised only at gauged points, the value that survives at the ungauged coordinates constituting *ColClim*’s primary use case is concentrated in the operational-scale evaluation itself (which establishes empirically, on ≈200,000 station–day observations, which distributional and covariate choices transfer to large, heterogeneous hydroclimatic data) and in the regional GAMM correction, which does something a station-level linear calibration cannot: it removes the cross-variable (Clausius–Clapeyron-coupled) relative humidity bias that the linear step leaves largely intact, and it is the only component that generalises to ungauged points where no station-specific calibration exists, with this spatial transfer being validated out-of-sample by leave-one-station-out cross-validation, including the spatio-temporal block design that withholds both the station and the difficult 2023–2025 window (Section 6.6). The modest, conditional joint gains are therefore best understood as a quantification of the exploitable cross-variable signal remaining in the marginal residuals a methodological characterisation rather than as the deployable headline figure.

Several methodological limitations merit explicit discussion. The spatial scope of the GMFAMM is restricted to the Valle del Cauca training network; predictions for points far from the 62 EMAs involve spatial extrapolation whose uncertainty is not currently quantified in the *ColClim* interface (a distance-to-nearest-station warning chip is planned for the next release).

The validation design in this paper is twofold. The primary hold-out is *temporal* (training on 2016–2022, testing on 2023–2025 at the same 62 EMAs), which assesses prospective predictive skill. To address spatial generalisation to ungauged locations, in the operational setting of *ColClim* we performed leave-one-station-out (LOSO) cross-validation in two designs (Section 6.6, Table 15), both in the genuinely ungauged configuration (station random intercept excluded, no station-specific calibration). An all-years design (median RMSE $0.96^{\;\circ }$C, $1.25^{\;\circ }$C, 3.90% for $T_{\min}$, $T_{\max}$, RH) isolates pure spatial transfer, and a spatio-temporal block design that withholds both the station and the difficult 2023–2025 window (median RMSE $1.19^{\;\circ }$C, $1.57^{\;\circ }$C, 4.55%) isolates spatial transfer on the same period as the temporal hold-out. The block error is essentially equal to the gauged temporal error for $T_{\min}$ and below it for $T_{\max}$ and RH, providing direct, leakage-free evidence that the regional correction transfers across space even under prospective conditions. Three honest limitations remain: the spatial analysis covers the three energy balance variables; relative humidity retains a long upper tail in the block design (P90 =8.46%), so accuracy at the poorly transferring minority of stations should not be over-stated; and a full distance-stratified error model and spatial-block cross-validation extended to solar radiation and precipitation occurrence are planned as the methodological focus of a follow-up paper. Practitioners using *ColClim* for points well beyond the training network’s spatial range (provisionally, more than ∼20 km from the nearest training station, pending the distance-stratified analysis identified above) should treat the corrected outputs as conditional bias-corrected estimates rather than locally validated observations; the planned distance indicator will make this boundary explicit in the interface.

The temporal stationarity assumption that the satellite-to-surface relationship estimated on 2016–2022 data generalises to future years may be challenged by strong ENSO events; periodic model retraining as new IDEAM data accumulates is planned. The solar radiation component is reported in kWh m^−2^ day^−1^; it remains the variable with the largest residual error after correction (≈2 kWh m^−2^ day^−1^), reflecting the intrinsic difficulty of correcting daily shortwave radiation in a region of strong convective cloudiness, and the absolute values should be interpreted with care in the absence of a dedicated pyrheliometer validation network. Finally, implementing the full simultaneous GMFAMM estimation (rather than the two-stage approximation) at the scale of ≈200,000 station–day observations remains a computational priority for future work; evaluating such a full joint model against the present two-stage approximation would benefit from purpose-built joint performance metrics for mixed-type multivariate responses, for which we have released an open-source R package (mvmetrics; see Data Availability) rather than the per-variable RMSE used here.

From the application perspective, the public instance of *ColClim* runs on a shared free-tier host; service credentials (for the optional AI-report module) are supplied through secure environment configuration rather than embedded in the application code, and any credential previously present in the source history has been revoked and rotated. Remaining steps toward a production-grade release include a caching layer for repeated NASA POWER queries and the parallelisation of multi-variable queries to reduce latency for extended date ranges.

## Figures and Tables

**Table 1:** Summary of station usage by analysis component. The five representative stations (*k*-means selection) are used only for visualisation; all quantitative evaluations use the full 62-EMA network.

Analysis Component	Stations	Purpose/Notes
Exploratory figures (Figure 2, Figure 3 and Figure 4, Appendix A)	5	Visualisation of representative climatic gradients
Model fitting (Section 5 and Section 6)	62	Station random intercepts estimated for all EMAs
Experimental grid evaluation (Table 6)	62	RMSE/LL averaged over all stations
Two-stage joint comparison (Table 9)	62 *	Out-of-sample metrics on 2023–2025 hold-out
Spatial LOSO validation (Table 15)	37 ^†^	Leave-one-station-out, ungauged configuration
Final performance tables (Table 10, Table 11, Table 12, Table 13 and Table 14)	37 ^†^	10th–90th percentile ranges across complete-case EMAs

* All 62 EMAs; the cross-variable comparison is additionally restricted to station–days on which all donor variables are jointly observed (n=36,727). ^†^ The 37 EMAs with complete-case records for all required covariates.

**Table 2:** Summary of NASA POWER reanalysis biases relative to surface observations (62 EMAs; 2016–2025). Bias = Reanalysis − Observed. The cold bias in temperature is associated with humidity overestimation, consistent with (though not in itself proof of) the Clausius–Clapeyron relation.

Variable	Obs. Mean	Sat. Mean	Mean Bias	RMSE	*r* (Daily)
$T_{\min}$ (°C)	18.93	13.85	−5.08	5.67	0.213
$T_{\max}$ (°C)	30.61	22.17	−8.44	8.86	0.504
RH (%)	80.70	85.20	+4.50	7.31	0.551
Rad (kWh/m^2^/day)	4.10	3.87	−0.23	1.38	0.621
$P_{\mathrm{bin}}$ (occ. rate)	52.2%	64.2%	+12.0 pp		0.309

For $P_{\mathrm{bin}}$ the bias is expressed in percentage points (pp): the reanalysis flags a rain event on 64.2% of days versus 52.2% observed, a +12.0 pp overestimation of rain day frequency. RMSE is not reported for the binary occurrence indicator.

**Table 3:** Study variables, their homologous NASA POWER predictor, and the distributional families screened in the experimental grid. The data type of each response (continuous, strictly positive continuous, or binary) is indicated in parentheses; the deployed (main) configuration selected for each variable is reported in Table 6.

Variable (Type)	NASA POWER Predictor	Families Evaluated
Min. Temperature ($T_{\min}$, continuous)	T2M_MIN	Gaussian, Gamma, Poisson
Max. Temperature ($T_{\max}$, continuous)	T2M_MAX	Gaussian, Gamma, Poisson
Relative Humidity (RH, continuous)	RH2M	Gaussian, Gamma, Poisson
Solar Radiation (Rad, positive continuous)	ALLSKY_SFC_SW_DWN	Gaussian, Gamma, Poisson
Precip. Occurrence ($P_{\mathrm{bin}}$, binary)	PRECTOTCORR	Binomial

Poisson is screened for the continuous variables only as a deliberate misspecification benchmark (Section 3.3); it is not a deployment candidate. The single configuration adopted per variable is given in Table 6.

**Table 4:** Summary of evaluated model configurations by response variable, distributional family, and effect structure.

Response	Family	Structure	Covariate Sets
$T_{\min}$	Gaussian	Linear, Smooth	8 sets each
	Gamma	Linear, Smooth	8 sets each
	Poisson	Linear, Smooth	8 sets each
$T_{\max}$	Gaussian	Linear, Smooth	8 sets each
	Gamma	Linear, Smooth	8 sets each
	Poisson	Linear, Smooth	8 sets each
RH	Gaussian	Linear, Smooth	8 sets each
	Gamma	Linear, Smooth	8 sets each
	Poisson	Linear, Smooth	8 sets each
Rad	Gaussian	Linear, Smooth	8 sets each
	Gamma	Linear, Smooth	8 sets each
	Poisson	Linear, Smooth	8 sets each
$P_{\mathrm{bin}}$	Binomial	Linear, Smooth	16 sets

Total: 224 model configurations.

**Table 5:** Out-of-sample RMSE (%) for relative humidity under the Gaussian, beta, and logit-normal families, refitted on the same optimal covariate set and chronological 70/30 hold-out (n=38,331 station–days). The Gaussian achieves the lowest error, supporting its selection.

Family	Link	RMSE (%)
Gaussian	identity	**5.08**
Beta	logit	5.14
Logit-normal	logit (Gaussian on logit scale)	5.20

Bold indicates the lowest RMSE among the three families evaluated.

**Table 6:** Configuration deployed for each response variable, selected for out-of-sample performance together with parsimony and physical interpretability (see text). RMSE for continuous variables; Log-Loss for $P_{\mathrm{bin}}$.

Response	Family	Structure	Key Covariates	RMSE/LL	MAPE (%)
$T_{\min}$ (°C)	Gaussian	Smooth	tmin_c + rad_kwh_m2	1.16	6.78
$T_{\max}$ (°C)	Gaussian	Smooth	tmax_c + rad_kwh_m2	1.63	4.49
RH (%)	Gaussian	Smooth	rh2m_pct + tmin_c + rad_kwh_m2	5.08	6.06
Rad (kWh/m^2^/day)	Gamma	Smooth	rad_kwh_m2 + rh2m_pct	2.10 ^†^	5.5
$P_{\mathrm{bin}}$	Binomial	Smooth	ppt_mm + rh2m_pct + tmin_c	0.3791 (LL)	

LL = Log-Loss. MAPE is not reported for $P_{\mathrm{bin}}$ (binary variable). AUC = 0.795 for the best precipitation occurrence model. ^†^ Rad RMSE in kWh m^−2^ day^−1^, on the same scale as in Table 9 and Table 13. For Rad, the Gamma and Gaussian families differ only marginally (Section 5); the joint comparison (Table 9) uses the Gaussian marginal on the matched subset—hence the small numerical difference. Solar radiation is the hardest variable to correct at daily resolution, consistent with the modest gains reported here.

**Table 7:** Sensitivity of the joint correction to the number of retained shared latent components $M_{0}$. Entries are the relative change in out-of-sample RMSE versus the marginal GAMM baseline (negative = improvement) on the 70/30 hold-out. Additional components yield further but modest out-of-sample reductions; $M_{0}=1$ is retained for parsimony and physical interpretability (see text). By construction the $M_{0}=1$ column coincides with the Joint-PC1 column of Table 9.

Variable	$M_{0}=1$	$M_{0}=2$	$M_{0}=3$
$T_{\min}$	−0.4%	−1.5%	−4.1%
$T_{\max}$	−16.6%	−19.2%	−19.5%
RH	−9.8%	−10.7%	−11.8%
Rad	−9.4%	−12.4%	−15.4%

All four variables improve with $M_{0}$; the gains beyond $M_{0}=1$ are small for $T_{\min}$, $T_{\max}$ and RH but appreciable for Rad (−9.4%→−15.4%). The $M_{0}=1$ column coincides with the Joint-PC1 result of Table 9. We default to $M_{0}=1$ for parsimony and physical/spatial transferability (PC2, PC3 lack a recognisable atmospheric interpretation, lie in the Kaiser ambiguity zone, and may not survive a spatial re-derivation), and expose $M_{0}$ as a configurable option so the gauged point accuracy/interpretability trade-off is left to the user (notably $M_{0}=3$ for Rad; see text).

**Table 8:** Eigenvalues and variance contributions from the PCA of the five-variable standardised residual matrix. PC1 is retained as the proxy for the dominant GMFAMM latent process $\Lambda_{0}\left(t\right)$.

Component	Eigenvalue	Variance Explained (%)	Cumulative (%)
PC1	1.924	38.49	38.49
PC2	1.049	20.97	59.46
PC3	1.019	20.38	79.85
PC4	0.575	11.49	91.34
PC5	0.433	8.66	100.00

**Table 9:** Out-of-sample RMSE of the marginal GAMM baseline and of the two-stage shared latent approximation (${\mathrm{Joint}}_{\mathrm{cross}}$: single donor residual; ${\mathrm{Joint}}_{\mathrm{PC}1}$: first principal component of the marginal residual matrix) on the chronological 70/30 hold-out (2023–2025). Δ% is the relative change versus the marginal baseline; negative = improvement. Bold marks the best joint variant per variable. The baseline is the deployed marginal model of Table 6 for every variable except Rad, for which the Gaussian marginal (within ≈0.5% of the deployed Gamma; Section 5) is used so that the shared latent step operates on a common Gaussian residual scale. Small numerical differences from Table 6 additionally arise because the joint comparison is restricted to station–days on which all donor variables are jointly observed (n=36,727).

Variable	Marginal	${\mathrm{Joint}}_{\mathrm{cross}}$	Δ%	${\mathrm{Joint}}_{\mathrm{PC}1}$	Δ%
$T_{\min}$ (°C)	1.154	1.138	−1.4%	1.149	−0.4%
$T_{\max}$ (°C)	1.607	1.539	−4.2%	**1.340**	−16.6%
RH (%)	5.087	**4.537**	−10.8%	4.588	−9.8%
Rad (kWh m^−2^ day^−1^)	2.086	1.955	−6.3%	**1.889**	−9.4%

All comparisons are made on the chronological 70/30 hold-out (2023–2025), and are *conditional on the donor variables being observed at the prediction location* (Section 6.3). They therefore quantify the exploitable cross-variable signal remaining in the marginal residuals; they are *not* predictive gains available at ungauged points, where the deployed pipeline applies the marginal correction only (validated spatially in Section 6.6). Both joint variants are leakage-free with respect to the recipient: ${\mathrm{Joint}}_{\mathrm{cross}}$ appends only a donor residual ($k^{\prime }\neq k$), and ${\mathrm{Joint}}_{\mathrm{PC}1}$ uses a leave-one-variable-out principal component that excludes the recipient’s own residual (Section 6.3). Rad is reported in kWh m^−2^ day^−1^; see Table 13 for the per-station performance of the deployed model. The shared latent step gives a modest, consistent benefit for Rad (≈9%; cf. Table 7).

**Table 10:** Deployed model performance for minimum temperature ($T_{\min}$). Ranges reflect the 10th–90th percentile across the 37 EMAs on the hold-out partition (2023–2025). Three correction stages are shown: raw NASA POWER reanalysis; station-level linear calibration only ($\hat{x}={\hat{\alpha }}_{i}+{\hat{\gamma }}_{i}x$, fitted on 2016–2022); and the full deployed two-stage correction.

Metric	NASA POWER (Raw)	Linear Corr. Only	Corrected (After)
RMSE (°C)	1.56–7.19	0.91–1.16	1.03–1.30
MAE (°C)	1.33–7.08	0.71–0.93	0.85–1.08
Mean Bias (°C)	−7.08 to −1.20	−0.40 to +0.03	−0.82 to −0.40
Pearson *r*	0.36–0.46		0.34–0.52

**Table 11:** Deployed model performance for maximum temperature ($T_{\max}$). Ranges reflect the 10th–90th percentile across the 37 EMAs. Station-level linear calibration alone removes 83% of the raw RMSE (median); the regional GAMM correction provides a further reduction. The incremental gain attributable specifically to the shared latent term is reported, on a like-for-like basis, in Table 9.

Metric	NASA POWER (Raw)	Linear Corr. Only	Corrected (After)
RMSE (°C)	6.24–10.50	1.51–1.77	1.46–1.75
MAE (°C)	6.01–10.37	1.18–1.40	1.19–1.46
Mean Bias (°C)	−10.37 to −6.00	−0.57 to −0.19	−1.10 to −0.76
Pearson *r*	0.62–0.70		0.74–0.81

**Table 12:** Deployed model performance for relative humidity (RH). Ranges reflect the 10th–90th percentile across the 37 EMAs. The modest gain of the linear correction alone (22.5% median RMSE reduction, vs. 83% for temperatures) reflects that the RH bias is driven by cross-variable Clausius–Clapeyron coupling rather than a simple additive offset; the regional GAMM correction and, where donor observations are available, the conditional shared latent term (Table 9) further reduces this bias.

Metric	NASA POWER (Raw)	Linear Corr. Only	Corrected (After)
RMSE (%)	4.30–10.12	4.25–5.70	3.29–5.24
MAE (%)	3.43–9.00	3.39–4.56	2.63–4.09
Mean Bias (%)	+0.70 to +8.80	−2.29 to +1.23	−0.80 to +2.92
Pearson *r*	0.58–0.71		0.71–0.82

**Table 13:** Deployed model performance for solar radiation (Rad), in kWh m^−2^ day^−1^. Ranges reflect the 10th–90th percentile across the 37 EMAs on the hold-out partition. Both the linear calibration and the GAMM reduce the raw error, the GAMM giving a further reduction; solar radiation is the hardest variable to correct at daily resolution.

Metric	NASA POWER (Raw)	Linear Corr. Only	Corrected (After)
RMSE (kWh m^−2^ day^−1^)	1.98–2.86	2.05–2.52	1.88–2.39
MAE (kWh m^−2^ day^−1^)	1.59–2.32	1.64–2.04	1.47–1.91
Mean Bias (kWh m^−2^ day^−1^)	−1.21 to +1.10	−1.01 to 0.00	−0.47 to +0.49
Pearson *r*	0.65–0.81		0.68–0.83

Values in kWh m^−2^ day^−1^. The per-station, test-period (2023–2025) ranges are larger than the pooled full-period raw RMSE of Table 2 (1.38), reflecting the harder hold-out years and the per-station spread. Both the linear calibration and the GAMM reduce the raw error, with the GAMM giving a further reduction; solar radiation remains the hardest variable to correct at daily resolution.

**Table 14:** Deployed model classifier performance for precipitation occurrence ($P_{\mathrm{bin}}$). Ranges reflect the 10th–90th percentile across the 37 EMAs. The linear correction on the raw ppt_mm covariate provides a modest baseline; the Binomial GAMM classifier improves accuracy and probabilistic calibration (lower Brier Score). Any additional shared latent (cross-variable) contribution for $P_{\mathrm{bin}}$ was not part of the reconciled like-for-like analysis of Section 6.3 and is treated as exploratory.

Metric	Raw NASA Event	Linear Corr. Only	Corrected (After)
Accuracy	0.71–0.79	0.74–0.79	0.78–0.86
Sensitivity (Recall)	0.68–0.76	0.64–0.73	0.76–0.87
Specificity	0.73–0.82	0.76–0.83	0.79–0.88
Precision	0.65–0.74	0.67–0.75	0.74–0.85
F1-Score	0.66–0.75	0.65–0.74	0.75–0.86
Brier Score	0.19–0.24	N/A ^†^	0.12–0.17

^†^ The linear correction maps a continuous rainfall amount directly to a probability without a logit link, yielding inflated Brier Scores. The GAMM Binomial model with proper probabilistic calibration resolves this. The per-station classifier metrics in all three columns are fully reproducible from the precipitation occurrence evaluation script in the openly available repository (see Data Availability), which regenerates the raw, linear-only, and corrected metrics from the IDEAM and NASA POWER inputs on the same chronological 70/30 hold-out.

**Table 15:** Temporal versus spatial out-of-sample RMSE for the marginal correction. *Temporal (62)*: chronological 70/30 hold-out at all gauged stations and gauged configurations (Section 6.3). *Temporal (37)*: the same gauged temporal hold-out recomputed on the 37 complete-case stations, shown to rule out a subset effect. *Spatial LOSO (all years)*: leave-one-station-out in the genuinely ungauged configuration (station random intercept excluded, no station-specific calibration), predicting each held-out station across all ten years. *Spatial LOSO (2023–2025 block)*: the same ungauged configuration trained on the remaining stations over 2016–2022 and evaluated on the held-out station over the difficult 2023–2025 window only, isolating spatial transfer on the same period as the temporal hold-out. Entries are the median [10th–90th percentile] across the 37 validated stations.

Variable	Temporal (62)	Temporal (37)	Spatial LOSO, All Years (Median [P10–P90])	Spatial LOSO, 2023–2025 Block (Median [P10–P90])
$T_{\min}$ (°C)	1.15	1.16	0.96 [0.88–1.01]	1.19 [0.96–1.61]
$T_{\max}$ (°C)	1.61	1.63	1.25 [1.12–1.49]	1.57 [1.25–1.78]
RH (%)	5.09	5.08	3.90 [3.59–4.76]	4.55 [3.50–8.46]

Gauged columns ("Temporal") use the deployed model with per-station calibration and random intercept; ungauged columns ("Spatial LOSO") use neither. Spatial validation is restricted to the three energy balance variables and to the 37 stations with complete-case records for the required covariates; performance is expected to be best within the convex hull of the training network and to degrade with distance, as flagged by the planned distance-to-nearest-station indicator (Section 7). The *"Block"* column withholds both the station and the 2023–2025 period, so it is directly comparable to the *"Temporal (37)"* column on the identical window.

**Table 16:** Summary of the five application tabs from the user perspective.

Tab	Purpose	Key Interaction	Data Source	Station?
Map	Geographic selection	Map click (snap ≤ 2 km)		No
Results	Visual exploration	Time scale (D/W/M/A)	NASA POWER	For valid.
Data Table	Review and download	CSV download; search	NASA POWER	No
AI Report	Interpretive analysis	AI report button	NASA POWER	Improves
Forecast	Short-range forecast	Horizon slider + variable	Open-Meteo	No

**Figure A1:**
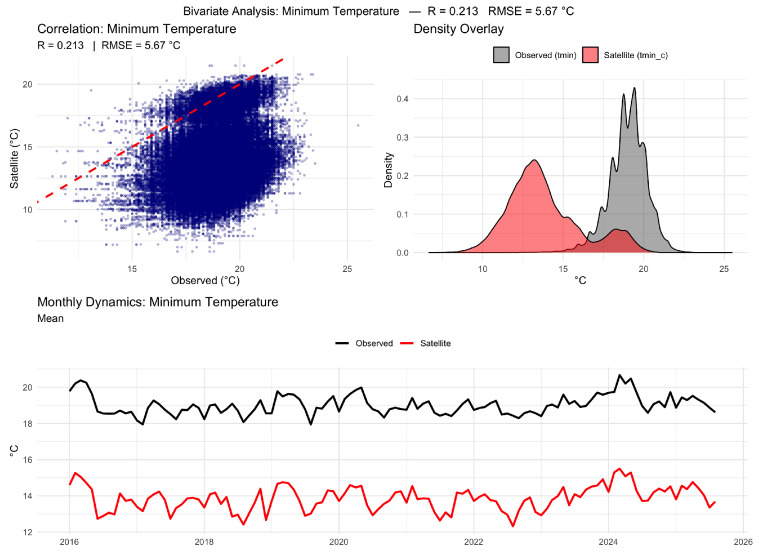
Bivariate analysis of minimum temperature (°C): R=0.213; $\mathrm{RMSE}=5.67^{\;\circ }$C. The scatter plot confirms the deep structural discrepancy; the monthly dynamics show a virtually parallel but offset pattern, confirming a purely additive cold bias of $5^{\;\circ }$C–$6^{\;\circ }$C throughout the study period.

**Figure A2:**
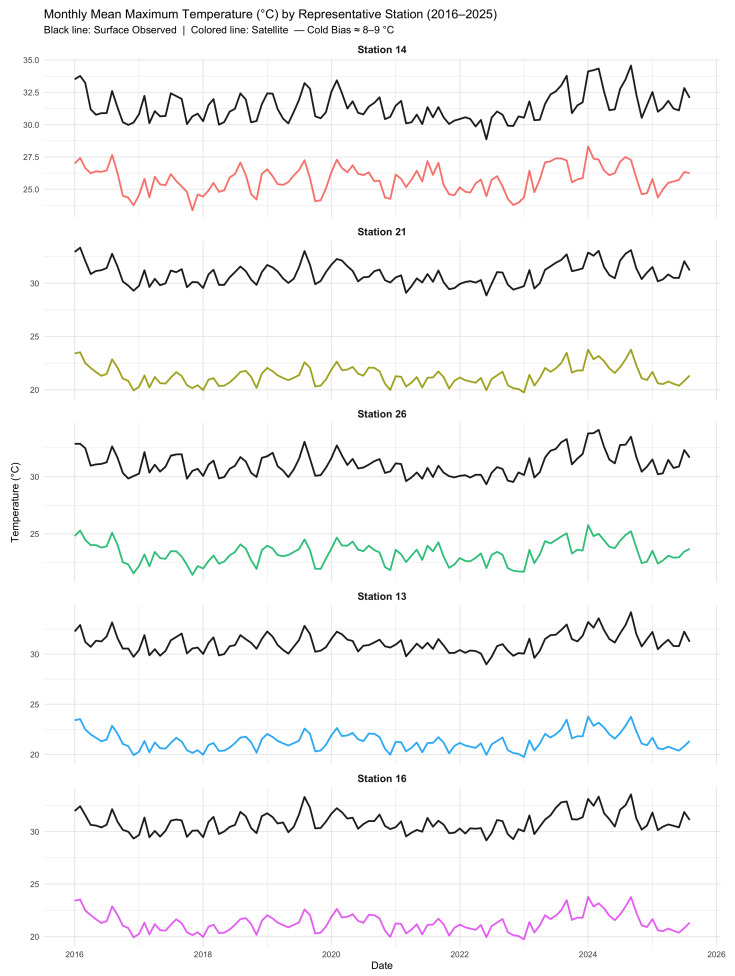
Monthly mean maximum temperature (°C) by representative station (2016–2025). Black line: observed. Colored line: satellite. The cold bias of ≈$8^{\;\circ }$C–$9^{\;\circ }$C is consistent and stable across all stations and throughout the entire period, confirming the additive and temporally stationary nature of the satellite error.

**Figure A3:**
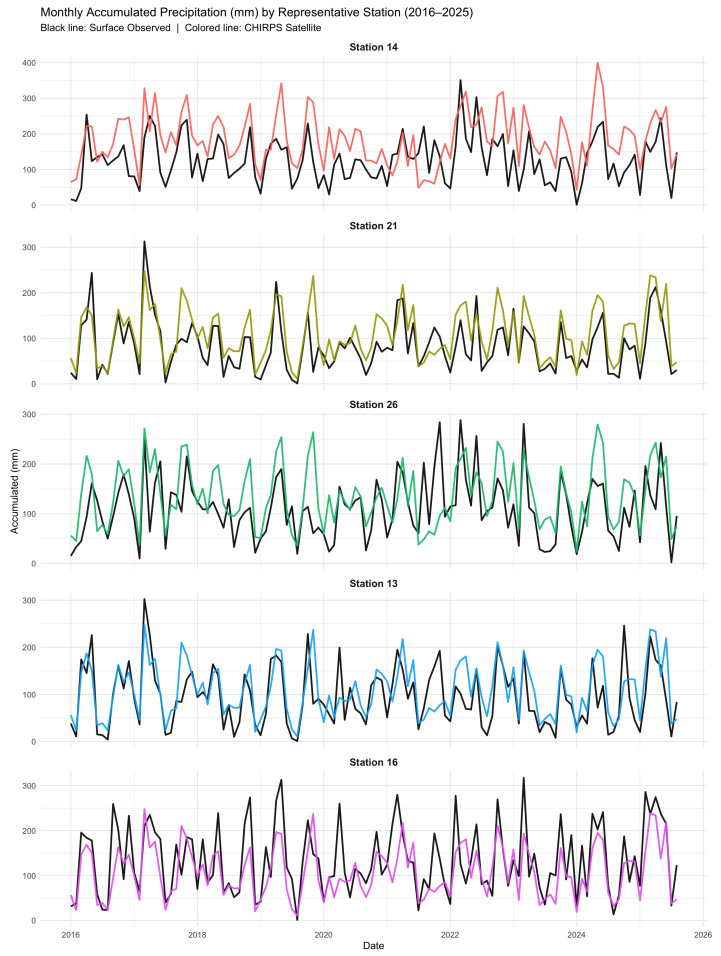
Monthly accumulated precipitation (mm) by representative station (2016–2025). Black line: observed. Colored line: satellite. Station 14 (north) exhibits the greatest overestimation of accumulated precipitation; station 13 (center) shows the best satellite–ground coupling.

**Figure A4:**
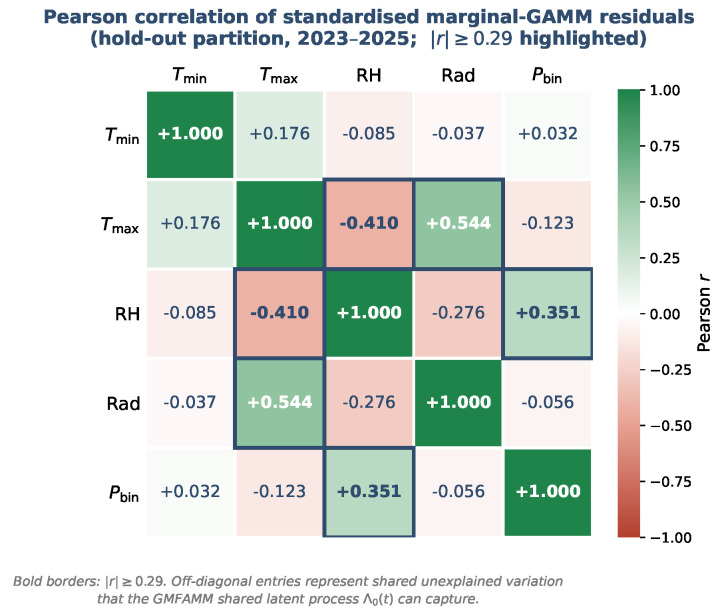
Pearson correlation matrix of standardised marginal GAMM residuals (**hold-out partition, 2023–2025**), for all five variables including precipitation occurrence ($P_{\mathrm{bin}}$). The two strongest training–partition pairs ($T_{\max}$–RH, $T_{\max}$–Rad) remain |r|>0.4: Rad–RH attenuates to r=−0.28 (just below the 0.29 outlining threshold) but stays significant (n≈36,000), while RH–$P_{\mathrm{bin}}$ (r=+0.351) is the binary outcome’s only outlined coupling; together these confirm that the cross-variable coupling is temporally stable and not a training artefact.

**Figure A5:**
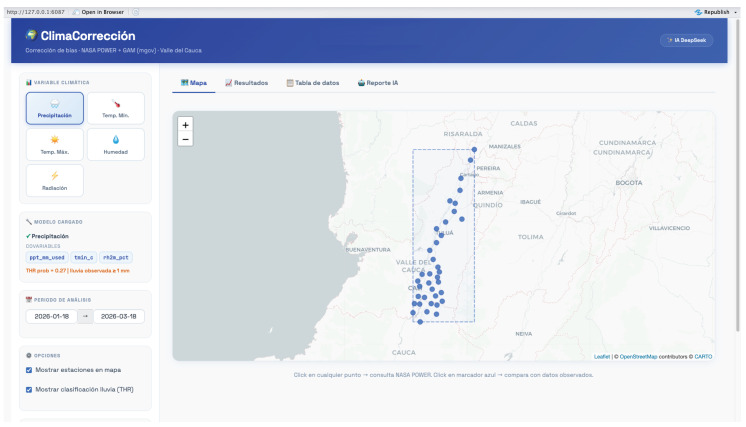
Live application Tab 1 (Interactive Map) with the precipitation model loaded. The sidebar shows the study zone selector, variable cards, and model information. The map renders two station layers: training stations (dark blue markers within the dashed domain rectangle) and observed stations colour-coded by variable type.

**Figure A6:**
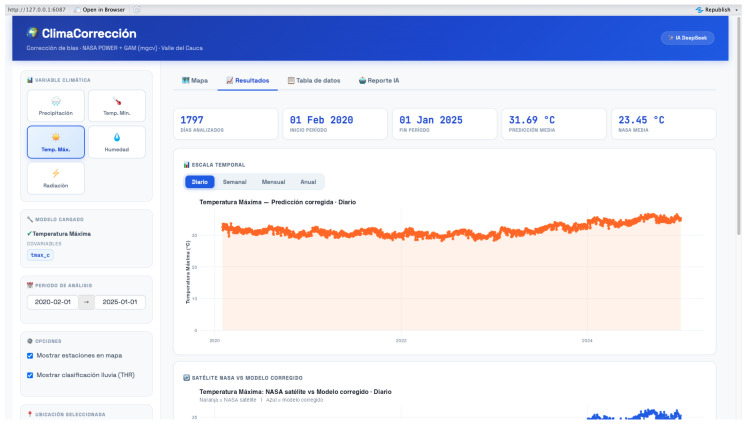
Live application Tab 2 (Results) for a Valle del Cauca station. The tripartite comparison chart shows: raw NASA POWER signal (orange, “Satélite crudo”), bias-corrected prediction (blue, “Corregido GAM”), and observed EMA station series (green, “Estación observada”). The correction substantially closes the gap between the satellite and ground truth for maximum temperature.

## Data Availability

The corrected analysis is fully reproducible. The evaluation and figure-generation scripts that regenerate every table and figure in this paper directly from the raw inputs including the correctly specified marginal baseline (Table 6), the corrected joint comparison (Table 9), the $M_{0}$ sensitivity (Table 7), the leave-one-station-out validation (Table 15), the per-station precipitation occurrence metrics (Table 14), and the bootstrap confidence intervals together with the *ColClim* application source code, are openly available at https://github.com/darango2025/ColClim (accessed on 6 June 2026). A live, publicly accessible instance of the application is hosted at https://4d4fmw-david-arango.shinyapps.io/ColClim/ (accessed on 6 June 2026). The NASA POWER reanalysis data can be accessed at https://power.larc.nasa.gov/ (accessed on 6 June 2026). The IDEAM surface station data that support the findings of this study are available upon request from the Instituto de Hidrología, Meteorología y Estudios Ambientales (IDEAM) of Colombia (https://www.datos.gov.co/Ambiente-y-Desarrollo-Sostenible/IDEAM/cqmv-a99d/about_data, accessed on 6 June 2026), subject to that institution’s data-sharing policy. The authors’ open-source R package mvmetrics, which implements joint performance metrics for mixed-type multivariate responses of the kind modelled here, is publicly available at https://github.com/darango2025/mvmetrics (accessed on 6 June 2026).

## Abbreviations

The following abbreviations are used in this manuscript:

GMFAMM	Generalized Multivariate Functional Additive Mixed Model
GAMM	Generalized Additive Mixed Model
FGAMM	Functional Generalized Additive Mixed Model
MFPCA	Multivariate Functional Principal Component Analysis
NASA POWER	NASA Prediction of Worldwide Energy Resources
EMA	Estación Meteorológica Automática (Automatic Meteorological Station)
IDEAM	Instituto de Hidrología, Meteorología y Estudios Ambientales
ENSO	El Niño–Southern Oscillation
NWP	Numerical Weather Prediction
RH	Relative Humidity
Rad	Solar Radiation
RMSE	Root Mean Square Error
MAE	Mean Absolute Error
MAPE	Mean Absolute Percentage Error
AUC	Area Under the ROC Curve
REML	Restricted Maximum Likelihood
KL	Karhunen–Loève
PC	Principal Component
PCA	Principal Component Analysis

## Appendix A. Supplementary Descriptive Analysis

Figure A1 shows the bivariate analysis for minimum temperature (R=0.213, $\mathrm{RMSE}=5.67^{\;\circ }$C), confirming a weak daily correlation and the point cloud as almost entirely below the identity line: the distributions are completely decoupled, with the satellite shifting mass from the observed $18^{\;\circ }$C–$20^{\;\circ }$C range to ≈$13^{\;\circ }$C.

Figure A2 shows the monthly time series for maximum temperature across the five representative stations. The cold bias of ≈$8^{\;\circ }$C–$9^{\;\circ }$C is consistent and stable across all stations and throughout the entire period.

Figure A3 shows the monthly accumulated precipitation by representative station. Station 14 (northern end of the valley) exhibits the greatest satellite overestimation; station 13 (center) shows the best coupling between satellite and observed series.

## Appendix B. Hold-Out Residual Correlation Matrix

For transparency, Figure A4 presents the Pearson correlation matrix of all five sets of standardised marginal GAMM residuals (including $P_{\mathrm{bin}}$) computed on the *hold-out* partition (2023–2025), complementing the training–partition matrix in Figure 8. The two strongest pairs ($T_{\max}$–RH and $T_{\max}$–Rad) remain well above |r|=0.4 on both partitions, and Rad–RH stays statistically significant (r=−0.28 on the hold-out, n≈36,000); the RH–$P_{\mathrm{bin}}$ coupling even strengthens slightly to r=+0.351 on the hold-out (cf. +0.291 in training), remaining above the 0.29 outlining threshold, confirming that the cross-variable dependence structure documented in Section 6.1 is not a training-set artefact.

## Appendix C. Application Interface Screenshots

For completeness, Figure A5, Figure A6 and Figure A7 illustrate the live *ColClim* interface referenced in Section 7: the interactive map for geographic selection (Tab 1), the tripartite results comparison (Tab 2), and the short-range forecast module (Tab 5). The interface header in these screenshots displays the application’s earlier working title (“ClimaCorrección”); the deployed, currently public instance is named *ColClim* throughout this manuscript.
